# Image-driven OCT calibration from a single B-scan

**DOI:** 10.1364/BOE.608117

**Published:** 2026-08-25

**Authors:** Owen Simmons, Philippe de Wilde, Adrian Podoleanu

**Affiliations:** 1 Applied Optics Group, University of Kent, Canterbury, Kent, CT2 7NH, UK; 2 Division of Natural Sciences, University of Kent, Canterbury, Kent, CT2 7NH, UK

## Abstract

Optical coherence tomography (OCT) requires accurate calibration of wavenumber sampling and dispersion compensation to achieve optimal axial resolution. Conventional calibration methods rely on dedicated mirror measurements acquired at multiple optical path differences, requiring dedicated acquisitions, careful alignment, and additional acquisition time. We present a physics-informed optimisation framework that recovers OCT calibration directly from a single routine B-scan. Within the complex leader-follower reconstruction framework, the method jointly estimates wavenumber remapping and dispersion compensation functions by optimising image quality in reconstructed OCT data. Experiments on a swept-source OCT system demonstrate that the recovered calibration functions produce image quality comparable to conventional mirror-based calibration while requiring only a single acquired image and 30–60 seconds of optimisation. Samples containing depth-distributed structure recover calibration functions closely matching the underlying system calibration, whereas structurally limited samples produce image-specific effective calibration within the observed depth range. These results demonstrate the feasibility of rapid software-defined OCT calibration directly from imaging data, reducing dependence on dedicated calibration measurements, and simplifying OCT system operation.

## 1. Introduction

Optical Coherence Tomography (OCT) is a depth-resolved interferometric imaging modality capable of micrometre-scale axial resolution [1,2]. In Fourier-domain OCT, this resolution depends critically on accurate reconstruction of the spectral interferogram as a function of optical wavenumber. Errors in spectral sampling or phase compensation directly degrade the axial point spread function (PSF), reducing image sharpness and limiting quantitative performance.

In spectral-domain OCT, the depth-resolved reflectivity profile A(z) is recovered from the spectral interferogram S(k) using a Fourier transform. Accurate reconstruction requires uniform sampling in wavenumber k . In practice, however, OCT systems acquire data non-uniformly in k -space. Spectrometer-based systems sample approximately uniformly in wavelength λ , while swept-source systems exhibit additional nonlinearities due to laser sweeping characteristics. As a result, the effective sampling function deviates from the assumed linear model. If uncorrected, this non-uniformity introduces frequency-domain chirp, producing axial broadening, sidelobes, and depth-dependent resolution loss.

Image quality is further degraded by dispersion imbalance between the interferometer reference and sample arms. This introduces a wavelength-dependent phase error into the interferogram, leading to PSF broadening and attenuation after reconstruction [3]. Together, wavenumber nonlinearity and dispersion produce composite distortions that degrade the depth-resolving capability of OCT and the resulting image quality.

Correcting these effects requires calibration of both the wavenumber sampling and dispersion compensation functions. Conventionally, this is achieved using interferograms acquired from a reference mirror positioned successively at several distances from the OCT interface optics, thereby providing a series of known optical path differences (OPDs) in the interferometer [4,5]. These measurements are then used to derive numerical resampling and phase compensation corrections prior to image reconstruction. While effective, mirror-based calibration requires dedicated acquisitions, careful alignment, and interruption of normal imaging workflows.

To reduce reliance on conventional mirror-based calibration, both hardware and computational alternatives have been explored. Hardware approaches include optical pre-linearisation with k-clocks [6] and intrinsic wavelength referencing using multi-line sources such as krypton lamps [7]. Computational approaches include numerical methods for dispersion compensation or wavenumber correction [8–11], as well as learning-based approaches that correct spectral nonlinearities directly from OCT data [12–14].

Within our research group, OCT reconstruction and calibration are performed using the Complex Leader-Follower (CLF) framework (originally termed Complex Master-Slave) [15]. In CLF, OCT calibration is represented by two functions, g(k) and h(k) , corresponding to wavenumber remapping and dispersion compensation, respectively. These functions fully define both CLF-based reconstruction and conventional FFT-based calibration pipelines [16]. In the conventional CLF calibration procedure, g(k) and h(k) are still recovered from dedicated mirror measurements acquired at multiple optical path differences.

An alternative approach is to estimate these calibration functions directly from OCT image data. A reliable image-driven calibration framework could simplify OCT workflows, reduce dependence on specialised calibration hardware, and enable rapid system verification using routine acquisitions.

In this work, we formulate OCT calibration as a physics-informed optimisation problem over the calibration functions g(k) and h(k) . Using the CLF reconstruction model, we directly optimise these functions to maximise image sharpness in reconstructed A-scans. The calibration functions are parameterised using adaptive cubic splines and estimated using a staged gradient-based optimisation procedure.

We evaluate the framework on multiple swept-source OCT datasets acquired from representative samples with varying structural characteristics. The results demonstrate that calibration derived from a single B-scan can achieve image quality comparable to conventional mirror-based calibration. Importantly, optimisation-derived solutions may correspond either to recovery of the underlying system calibration or to an image-specific effective calibration, depending on the structural diversity present within the sample.

## 2. Theory

### 2.1. Complex leader-follower framework as a calibration model

In the CLF framework, OCT calibration is represented by two functions, g(k) and h(k) , describing wavenumber remapping and dispersion compensation, respectively. This section formalises their relationship to the measured interferometric phase and to OCT image reconstruction.

The phase evolution of the spectral interferogram from a single reflector forms the basis of OCT calibration. For a reflector at physical depth z , the interferometric phase evolves linearly with the true optical wavenumber $k_{phys}$ :

(1) $$\phi (k_{phys};z)=2zk_{phys}+dispersion+\phi_{\mathrm{const}},$$

In practical OCT systems, however, the measured spectrum is not sampled uniformly in $k_{phys}$ . Spectrometer-based systems sample approximately uniformly in wavelength, while swept-source systems sample according to the temporal tuning characteristics of the laser. In both cases, the relationship between acquisition index and physical wavenumber is nonlinear.

The Complex Leader–Follower (CLF) framework models the measured spectral phase directly in acquisition coordinates. For a reflector at an OPD 2z , the phase can be written as:

(2) $$\phi (k;z)=2z\cdot g(k)+h(k)+\phi_{\mathrm{rand}},$$

where:

•
ϕ(k;z) is the unwrapped spectral phase expressed in the sampled coordinate,
•
g(k) is a monotonic mapping function that encodes the effective wavenumber coordinate associated with each spectral sample,
•
h(k) captures phase distortions arising from dispersion imbalance between the interferometer arms,
•
$\phi_{\mathrm{rand}}$ is a depth-dependent constant phase offset caused by sub-wavelength path fluctuations.

In this representation, g(k) and h(k) jointly characterise the calibration state of the system.

Calibration therefore consists of estimating the functions g(k) and h(k) that restore the expected linear phase–depth relationship in the effective reconstruction coordinate [15].

Differentiating with respect to the sampled coordinate eliminates the constant phase term:

(3) $$\frac{\partial \phi (k;z)}{\partial k}=2z\cdot \frac{\partial g(k)}{\partial k}+\frac{\partial h(k)}{\partial k}.$$

Acquiring interferograms from reflectors at multiple depths yields a linear system in z, enabling recovery of $g^{\prime }(k)$ and $h^{\prime }(k)$ , and hence g(k) and h(k) up to physically irrelevant constants.

Once determined, g(k) and h(k) enable image reconstruction through the CLF method. For any depth z , a complex reference mask is constructed as:

(4) $$M_{z}(k)=\exp\left\{-i\left[g(k)\cdot 2z+h(k)\right]\right\}.$$

The A-scan is then computed by correlating the experimental spectrum S(k) with these masks:

(5) $$A(z)=\sum_{k}S(k)\cdot M_{z}^{*}(k)=\sum_{k}S(k)\;\exp\left\{i\left[g(k)\cdot 2z+h(k)\right]\right\}.$$

Equation (5) reveals that an image A(z) depends explicitly on the acquired data and on g(k) and h(k) . This dependency suggests an alternative approach to calibration: rather than deriving g and h from mirror measurements, one can instead search for the functions that optimise a suitable image-quality objective. This lets us reformulate calibration as an optimisation problem:

(6) $$(g_{\mathrm{opt}},h_{\mathrm{opt}})=\underset{g,h}{\arg\min}\;L\left(A(z;g,h)\right),$$

where L is a measure to quantify the quality and calibration of the image.

The remainder of this paper develops a practical algorithm to solve this optimisation problem. The key challenges are to parameterise the functions g(k) and h(k) effectively and to design a loss function L and optimisation strategy that robustly converges to the system calibration. These are addressed in the following Methods section.

## 3. Methods

### 3.1. Mathematical framework and optimisation algorithm

The core aim of this work is to estimate the calibration functions g(k) and h(k) directly from OCT image data without requiring dedicated mirror measurements. This is achieved by parameterising the calibration functions and optimising their parameters to maximise reconstructed image quality. The framework consists of three main components: (i) a parameterisation of g(k) and h(k) , (ii) an image-quality loss function, and (iii) an optimisation strategy for estimating the parameters.

#### 3.1.1. Parameterisation of calibration functions

##### Wavenumber Nonlinearity g(k)

The function g(k) effectively maps from the sampled spectral coordinates to the physical wavenumber. The precise form of this function varies between OCT systems and can exhibit complex, irregular shapes. Rather than assuming a specific analytic form, we seek a flexible representation that can adapt to these variations while respecting known physical constraints. The calibration function is expected to vary smoothly across the spectral range while preserving the ordering of spectral samples.

We therefore represent g(k) as a perturbation from a linear baseline:

(7) $$g(k)=g_{\mathrm{linear}}(k)+\Delta g(k),$$

where $g_{\mathrm{linear}}(k)$ represents uniform wavenumber sampling and Δg(k) describes the system-specific deviation from this baseline.

The correction Δg(k) is represented by a natural cubic spline interpolant defined by $N_{g}$ control points distributed across the spectral range. The values of the spline at the control-point locations are treated as optimisable parameters, while the correction is fixed to zero at the two endpoints. Between control points, Δg(k) is evaluated as a piecewise cubic polynomial with continuous first and second derivatives. Natural boundary conditions are imposed by setting the second derivative of the spline to zero at both endpoints.

This representation provides local flexibility to model system-specific wavenumber nonlinearities while maintaining a smooth calibration function and substantially reducing the number of optimisable parameters compared with independently optimising every spectral sample [17].

##### Dispersion Compensation: h(k)

Dispersion imbalance in OCT arises from differences in group velocity dispersion between the interferometer’s reference and sample arms. This occurs when optical materials (e.g., glass, biological tissue) or path lengths differ between arms, causing wavelength-dependent phase shifts. The function h(k) characterises this net phase error across the spectrum.

Unlike the nonlinearity captured by g(k) , dispersion follows a predictable physical structure in theory [8]. For most OCT systems, the phase error can be well-approximated by a Taylor expansion around a central wavenumber $k_{0}$ :

(8) $$h_{\mathrm{phys}}(k)\approx a_{0}+a_{1}(k-k_{0})+a_{2}{\left(k-k_{0}\right)}^{2}+a_{3}{\left(k-k_{0}\right)}^{3}+\cdots .$$

The constant and linear terms correspond respectively to a global phase offset and an axial image shift and therefore do not contribute to axial broadening. The dominant contribution to dispersion-induced resolution loss is consequently represented by the quadratic term [8]. We therefore use a quadratic baseline,

(9) $$h_{\mathrm{baseline}}(k)=a_{2}{\left(k-k_{0}\right)}^{2},$$

where $a_{2}$ is an optimisable parameter.

To allow deviations from an ideal quadratic dispersion model, the baseline is augmented by a smooth correction,

(10) $$h(k)=h_{\mathrm{baseline}}(k)+\Delta h(k),$$

where Δh(k) is represented using the same natural cubic spline interpolation used for Δg(k) . The spline is defined by $N_{h}$ optimisable control-point values, with zero endpoint corrections and natural boundary conditions.

This hybrid parameterisation incorporates the expected quadratic structure of the dispersion phase while retaining sufficient flexibility to represent smooth system-specific residuals. The spline representation also limits the dimensionality of the optimisation and discourages rapid, non-physical variations in the recovered calibration function.

#### 3.1.2. Loss function design

The optimisation framework requires a differentiable loss function L that quantifies the calibration of an image and provides a useful gradient signal for estimating g(k) and h(k) .

A natural objective for OCT calibration is the recovery of optimal axial resolution. In principle, this could be measured directly via the full width at half maximum (FWHM) of isolated point spread functions (PSFs). However, this approach is not applicable in the present setting: prior to calibration, phase errors cause significant axial broadening and overlap of reflectors, making reliable peak identification and resolution measurement infeasible.

Instead, we seek a surrogate objective based on the expected structural properties of well-calibrated OCT data. In a correctly calibrated system, isolated reflectors produce compact axial responses approaching the system PSF. Miscalibration spreads this energy axially, reducing sparsity in the reconstructed A-scan [18].

This observation motivates the use of sparsity as a proxy for image quality. We quantify sparsity using the Hoyer measure [19], a bounded and scale-invariant metric widely used in sparse signal processing.

For a normalised A-scan $\hat{A}(z)$ sampled at $N_{z}$ axial positions, the Hoyer sparsity is defined as

$$S(\hat{A})=\frac{\sqrt{N_{z}}-\frac{\|\hat{A}{\|}_{1}}{\|\hat{A}{\|}_{2}}}{\sqrt{N_{z}}-1},$$

where

$$\|\hat{A}{\|}_{1}=\sum_{z}|\hat{A}(z)|,\;\|\hat{A}{\|}_{2}=\sqrt{\sum_{z}{\left|\hat{A}(z)\right|}^{2}}.$$

$S(\hat{A})$ lies in the interval [0,1] , with S=1 corresponding to maximal sparsity (all energy concentrated into a single depth) and S=0 corresponding to a uniform distribution. Higher sparsity therefore corresponds to stronger axial localisation of signal energy, which is generally associated with improved effective axial resolution.

The sparsity-based loss for a single A-scan is defined as

$$L_{\mathrm{sparse}}=-S(\hat{A}).$$

The negative sign ensures that minimisation of the loss promotes concentration of energy into well-localised reflectors.

Alternative image-quality metrics, including entropy-based and statistical measures, were explored during preliminary experiments. In practice, these metrics either produced less stable optimisation behaviour or showed weaker correspondence with axial localisation of reflector energy. Hoyer sparsity was therefore selected as a simple, bounded, and differentiable objective that consistently promoted compact axial structure during optimisation. Because image sharpness alone does not uniquely constrain the underlying calibration functions, the optimisation may admit multiple locally optimal solutions depending on the structural content of the sample.

In addition to the image-based objective, regularisation is required to ensure that the recovered calibration functions remain physically plausible. Both g(k) and h(k) arise from smooth optical processes and are therefore expected to vary smoothly with wavenumber. Without constraint, the spline parameterisation may introduce high-frequency oscillations that improve the sparsity objective without corresponding to physically meaningful calibration functions.

To enforce smoothness, we include a second-order Tikhonov regularisation term [20]:

(11) $$L_{\mathrm{smooth}}={\left\|\frac{d^{2}g}{dk^{2}}\right\|}^{2}+{\left\|\frac{d^{2}h}{dk^{2}}\right\|}^{2}.$$

where the derivatives are evaluated numerically across the sampled spectral coordinate. For a B-scan comprising $N_{A}$ A-scans, the total loss is defined as

(12) $$L_{\mathrm{total}}=\frac{\lambda_{\mathrm{sparse}}}{N_{A}}\sum_{n=1}^{N_{A}}L_{\mathrm{sparse}}^{\left(n\right)}+\lambda_{\mathrm{smooth}}L_{\mathrm{smooth}},$$

where $\lambda_{\mathrm{sparse}},\lambda_{\mathrm{smooth}}>0$ are controllable weighting terms.

The weighting parameters were selected empirically during preliminary experiments. $\lambda_{\mathrm{sparse}}$ was fixed to 1, while $\lambda_{\mathrm{smooth}}$ was chosen to be the smallest value that consistently suppressed non-physical oscillations without noticeably restricting optimisation performance. The same weighting parameters were then used for all datasets.

#### 3.1.3. Optimisation strategy

The calibration parameters are estimated using gradient-based optimisation implemented in PyTorch. Automatic differentiation enables efficient computation of gradients through the differentiable reconstruction pipeline.

The parameters defining g(k) and h(k) are optimised jointly, since both functions contribute simultaneously to the reconstructed spectral phase and are therefore coupled through the image-quality objective. Preliminary experiments with sequential optimisation did not provide a consistent improvement in convergence or reconstruction quality, and simultaneous optimisation was therefore retained.

**Fig. 1.**
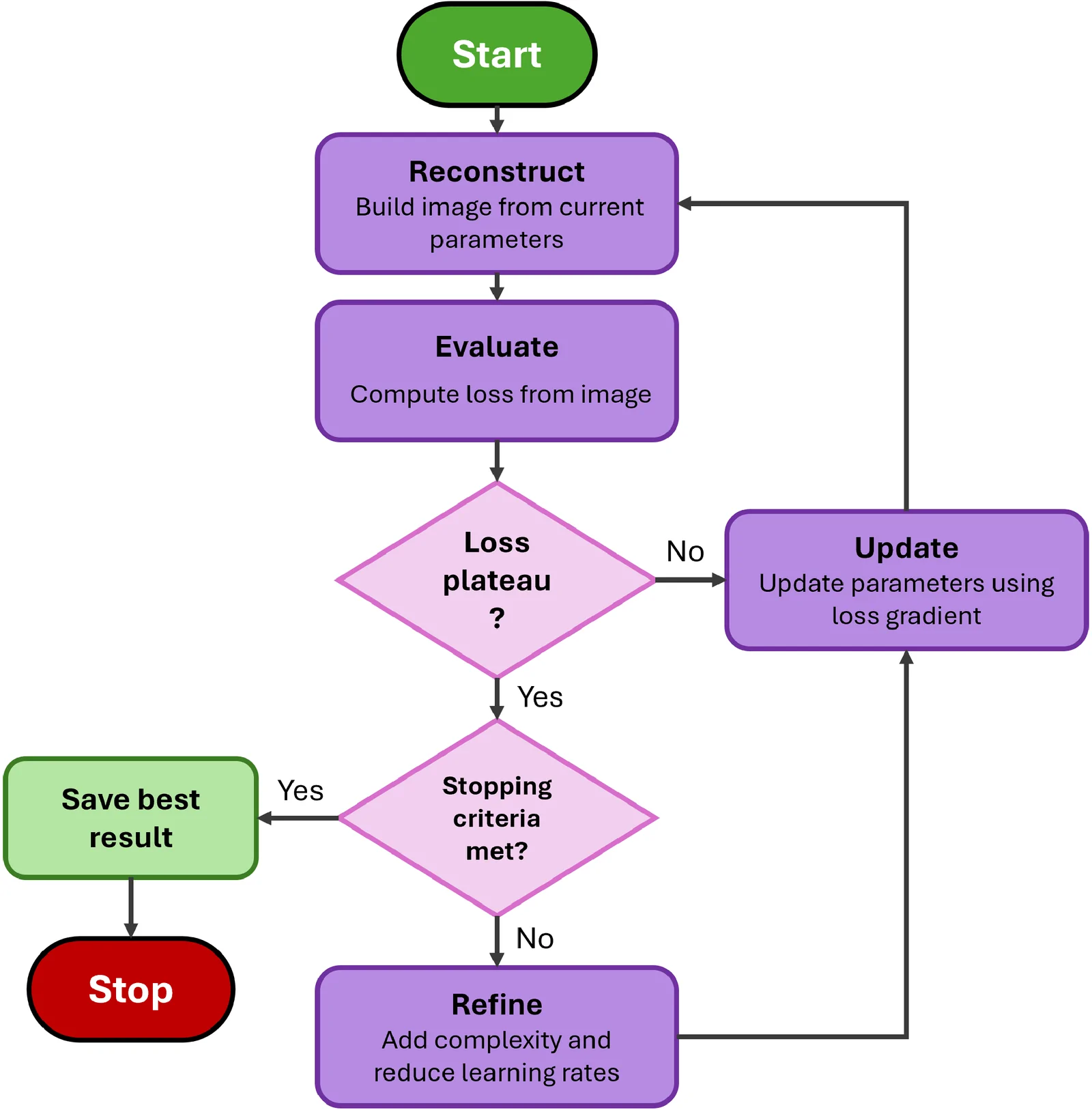
Schematic illustration of the staged optimisation strategy. The algorithm begins with a small number of spline control points for g(k) and h(k) , then progressively increases the spline resolution as optimisation converges.

The overall optimisation procedure is illustrated schematically in Fig. 1.

##### Progressive Model Refinement

Direct optimisation of a high-dimensional spline representation can lead to unstable convergence and sensitivity to local minima. To improve robustness, the optimisation proceeds through multiple stages with progressively increasing spline resolution.

The optimisation is initialised using a small number of spline control points, restricting the model to large-scale corrections of the calibration functions. Once convergence is reached at a given stage, additional control points are activated, and optimisation continues from the previous solution. Progression between refinement stages is triggered when the loss plateaus over a predefined number of successive optimisation iterations. This coarse-to-fine strategy stabilises the optimisation by recovering the global structure of the calibration functions before introducing higher-frequency degrees of freedom.

##### Computational Details

All optimisation experiments were performed using the Adam optimiser. For a single B-scan comprising 200 A-scans with 2048 spectral samples each, the complete optimisation typically required 30–60 seconds on an NVIDIA RTX 4070 GPU. CPU execution required approximately twice as long. Runtime per iteration increased during the progressive refinement stages as additional spline control points were introduced and the number of optimisable parameters increased. Consequently, the later, higher-resolution spline stages accounted for a greater proportion of the total optimisation time than the initial coarse stages. Increasing the number of A-scans used in the image-quality calculation produced a comparatively minor increase in runtime.

### 3.2. Experimental methods

#### 3.2.1. OCT system and datasets

Experiments were performed using an Applied Optics Group-built swept-source OCT system operating at a central wavelength of 1060 nm with a bandwidth of 100 nm.

**Fig. 2.**
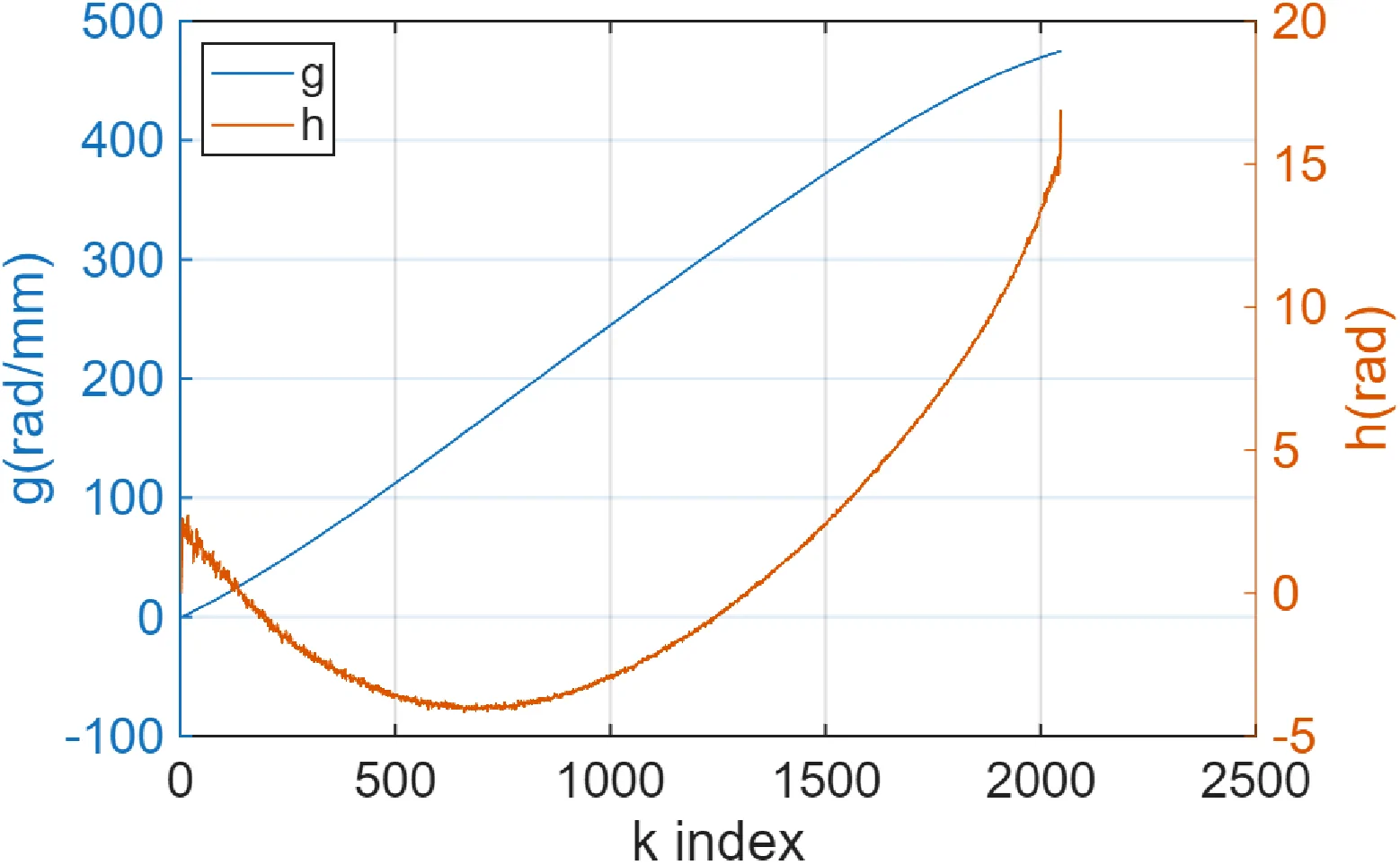
Reference calibration functions g(k) and h(k) obtained using conventional mirror-based calibration.

Reference calibration functions g(k) and h(k) were obtained using a conventional mirror-based calibration procedure and were used for comparison with the optimisation-derived calibration functions. The recovered reference functions are shown in Fig. 2.

To evaluate the optimisation framework under different structural imaging conditions, B-scans were acquired from four representative samples:

• **Sellotape phantom**: multiple layers of sellotape alternately stacked with annular thin scattering layers of paper, producing well-separated reflective interfaces distributed across depth.
• **IR card**: a standard infrared imaging card containing spatially heterogeneous scattering structure.
• ***In vivo* finger**: *in vivo* tissue data containing spatially continuous scattering features.
• **Paper**: a structurally limited sample dominated by a single reflective interface.

These datasets were selected to provide varying degrees of axial structural diversity for evaluating calibration recovery.

For all experiments, the optimisation algorithm operated directly on the raw uncalibrated spectral interferograms. No prior calibration information or mirror measurements were provided during optimisation.

#### 3.2.2. Evaluation metrics

Performance was evaluated using both image-based metrics and direct comparison with the reference calibration functions.

**Image-based Evaluation** For each dataset, optimisation-derived calibration functions g(k) and h(k) were used to reconstruct corresponding B-scans. Reconstructions were compared against both uncalibrated images and images reconstructed using conventional mirror-based calibration.

Axial resolution was quantified using the full width at half maximum (FWHM) of reconstructed axial peaks. For each A-scan, the dominant peak was identified and its FWHM measured. The mean FWHM across all A-scans within the B-scan was then calculated.

**Calibration-function Evaluation** Recovered calibration functions were compared with the reference mirror-derived calibration through analysis of their derivatives $g^{\prime }(k)$ and $h^{\prime }(k)$ . The derivatives provide a sensitive comparison of local functional agreement between the recovered and reference functions.

To ensure a consistent convention for the dispersion calibration, the reference h(k) was first transformed to the same zero-linear convention as the optimisation by removing its first-order Taylor component about the central wavenumber.

Prior to differentiation, the calibration functions were smoothed using a low-pass filter to suppress high-frequency numerical fluctuations. Agreement between the optimisation-derived and reference derivatives was quantified using normalised root-mean-square error (NRMSE) and cosine similarity [21]. NRMSE quantifies the magnitude of the difference relative to the variation in the reference function, while cosine similarity measures similarity in functional shape.

Calibration performance was additionally evaluated across imaging depth using mirror interferograms acquired at ten OPDs. Each interferogram was reconstructed using the mirror-derived reference calibration and each optimisation-derived calibration, producing an A-scan representing the system PSF at that OPD. The axial FWHM of each PSF was then measured as a function of depth. Consistent axial resolution across OPD indicates recovery of calibration functions that generalise beyond the depth distribution of the sample used for optimisation.

## 4. Results

### 4.1. Optimisation behaviour

**Fig. 3.**
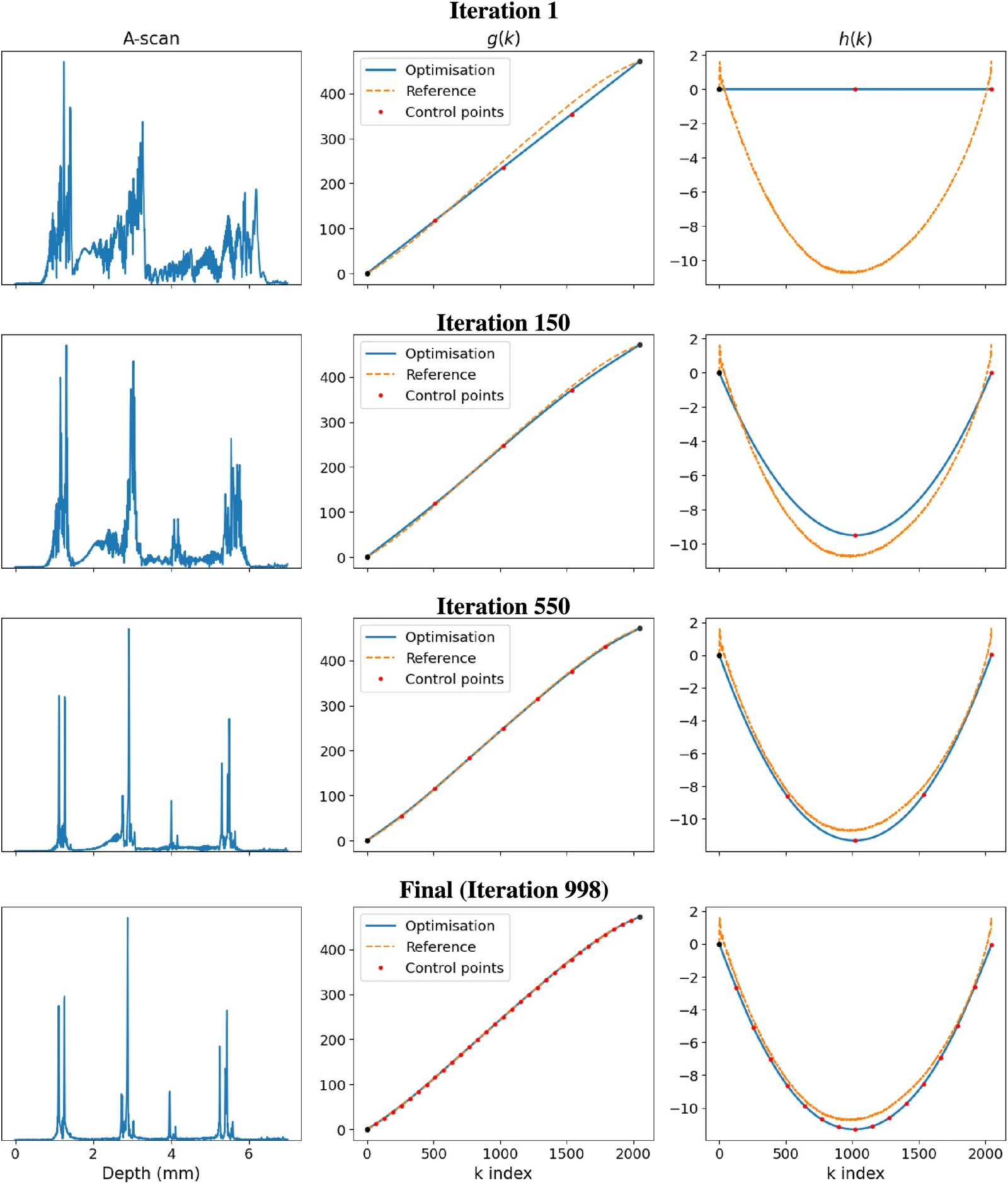
Visualisation of the optimisation process for the sellotape phantom dataset. The red points indicate spline control points used to parameterise the calibration functions. During optimisation, these control points are iteratively adjusted until the reconstructed image quality is maximised.

Figure 3 illustrates the staged optimisation procedure for the sellotape phantom. The optimisation is initialised using a coarse spline parameterisation, restricting the calibration functions to low-frequency corrections. As optimisation progresses, additional spline control points are introduced, enabling progressively finer adjustments to g(k) and h(k) .

Across successive optimisation stages, the reconstructed image exhibits increasing axial localisation and improved interface sharpness. The optimisation converged consistently for all evaluated datasets.

### 4.2. Image quality improvement

**Fig. 4.**
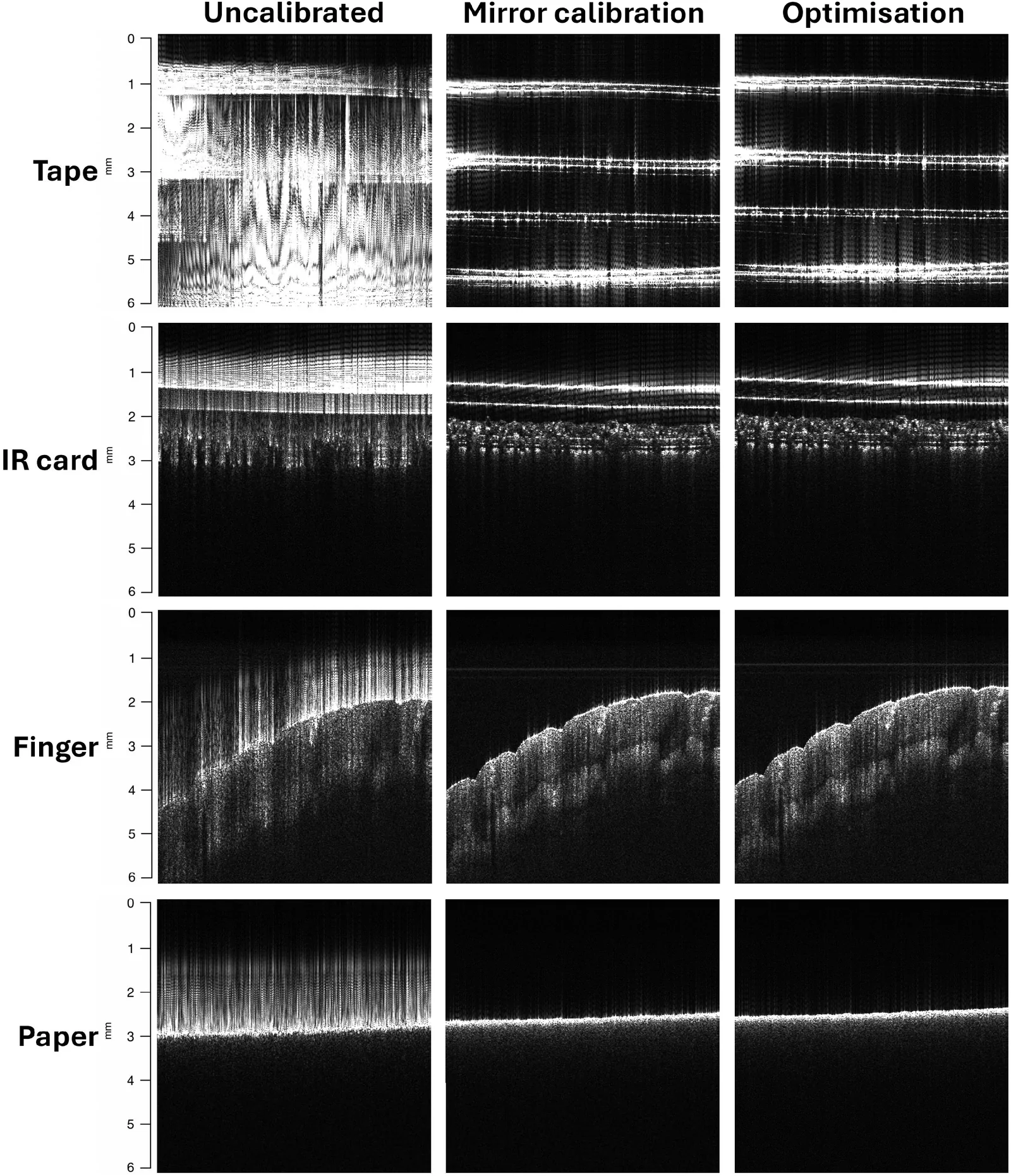
Example B-scan reconstructions for four representative samples. No spectral apodisation was applied in order to visualise the full effect of the recovered calibration functions.

The effectiveness of the optimisation method on real OCT images is illustrated in Fig. 4. Each row shows reconstructions for a different sample using three calibration conditions: an uncalibrated reconstruction, reconstruction using conventional mirror calibration, and reconstruction using the optimisation-derived calibration.

Across all samples, the optimisation procedure produces a clear improvement in axial sharpness relative to the uncalibrated reconstruction. The optimisation-derived images exhibit comparable sharpness relative to the mirror-calibrated images.

These qualitative observations are supported by measurements of reflector localisation within the reconstructed B-scans. Table 1 reports the mean FWHM of the dominant peak in each A-scan for both the optimisation-derived and mirror-based calibrations.

**Table 1.** Axial FWHM ( μ m) measured from reconstructed B-scans using mirror calibration and optimisation-derived calibration. Values are reported as mean ± standard deviation across all A-scans.

**Sample**	**Reference resolution**	**Optimised resolution**
Sellotape	22.7 ± 2.1	22.3 ± 2.2
IR card	21.2 ± 2.1	21.2 ± 2.2
Finger	27.0 ± 8.8	26.7 ± 8.2
Paper	29.5 ± 10.1	30.2 ± 10.5

Across all datasets, the optimisation-derived calibration produced mean FWHM values closely matching those obtained using mirror-based calibration. The largest discrepancies were below 1μm , substantially smaller than the observed variability within each dataset. Increased variance was observed for the finger and paper datasets, reflecting the broader and less well-defined reflector structure present in these samples.

### 4.3. Calibration comparison

**Fig. 5.**
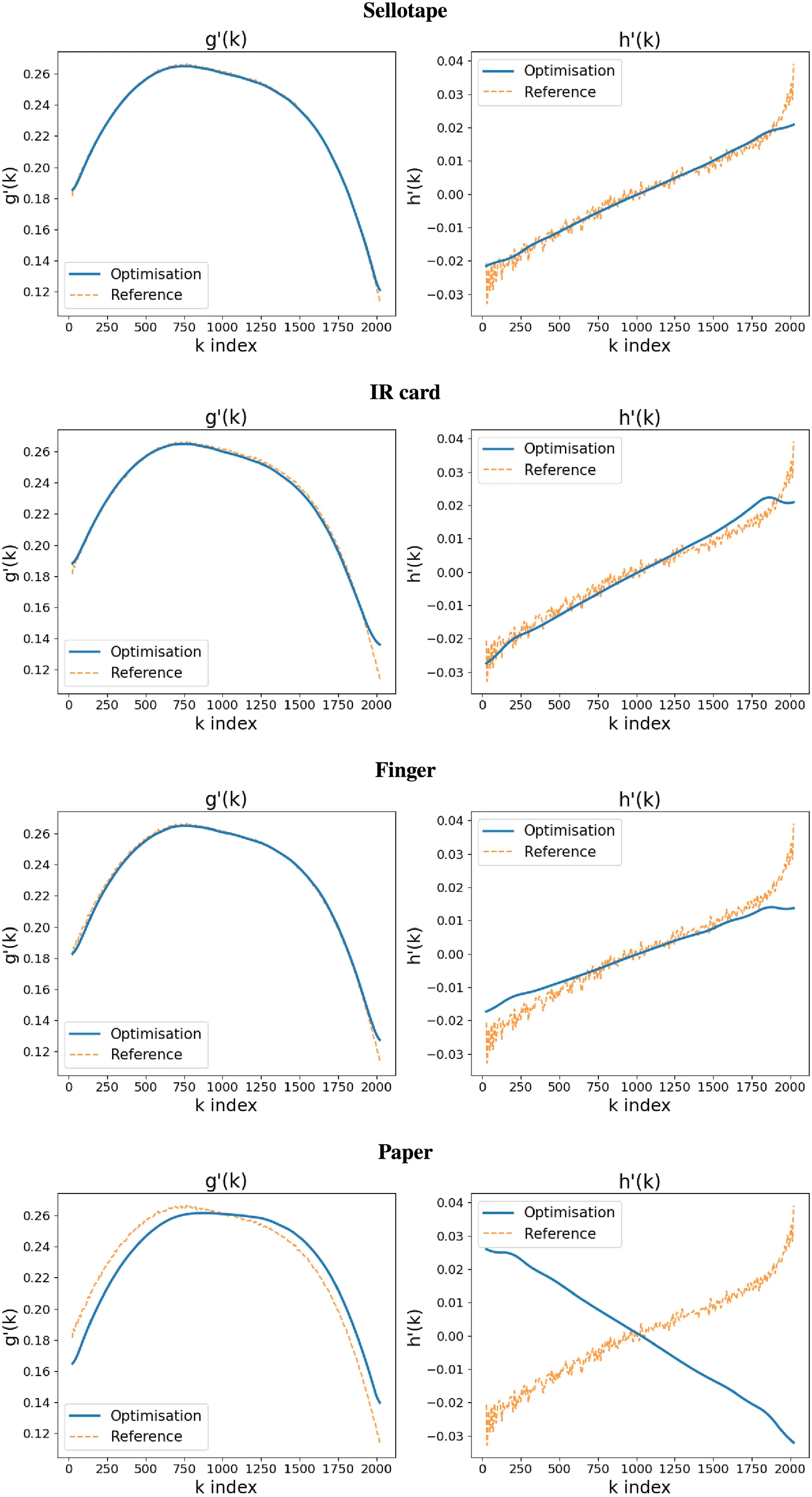
Comparison between the derivatives of the calibration functions recovered by the optimisation procedure using each sample and those recovered from standard mirror-based calibration.

To assess recovery of the underlying calibration functions, the smoothed derivatives of the optimisation-derived g(k) and h(k) were compared with those obtained from conventional mirror calibration. Figure 5 shows the comparisons for all four datasets.

The sellotape phantom, IR card and *in vivo* finger datasets show close overall agreement with the reference derivatives, with modest sample-dependent differences visible in both functions. The paper dataset diverges substantially, with $g^{\prime }(k)$ showing considerable deviation from the reference and $h^{\prime }(k)$ converging to an opposing trend. Quantitative agreement is summarised in Table 2 using NRMSE and cosine similarity.

**Table 2.** Normalised root-mean-square error and cosine similarity between the smoothed derivatives of the optimisation-derived and reference calibration functions.

**Sample**	**NRMSE $g^{\prime }(k)$ **	**NRMSE $h^{\prime }(k)$ **	**CosSim $g^{\prime }(k)$ **	**CosSim $h^{\prime }(k)$ **
Sellotape	0.0270	0.177	1.000	0.985
IR card	0.0843	0.219	0.998	0.983
Finger	0.0585	0.351	0.999	0.982
Paper	0.318	2.230	0.940	-0.987

The table reflects the trends visible in Fig. 5. The sellotape phantom gives the closest agreement overall, while the paper dataset is the clear outlier. Among the remaining datasets, the *in vivo* finger and IR card show similarly strong agreement, with slight differences between $g^{\prime }(k)$ and $h^{\prime }(k)$ .

**Fig. 6.**
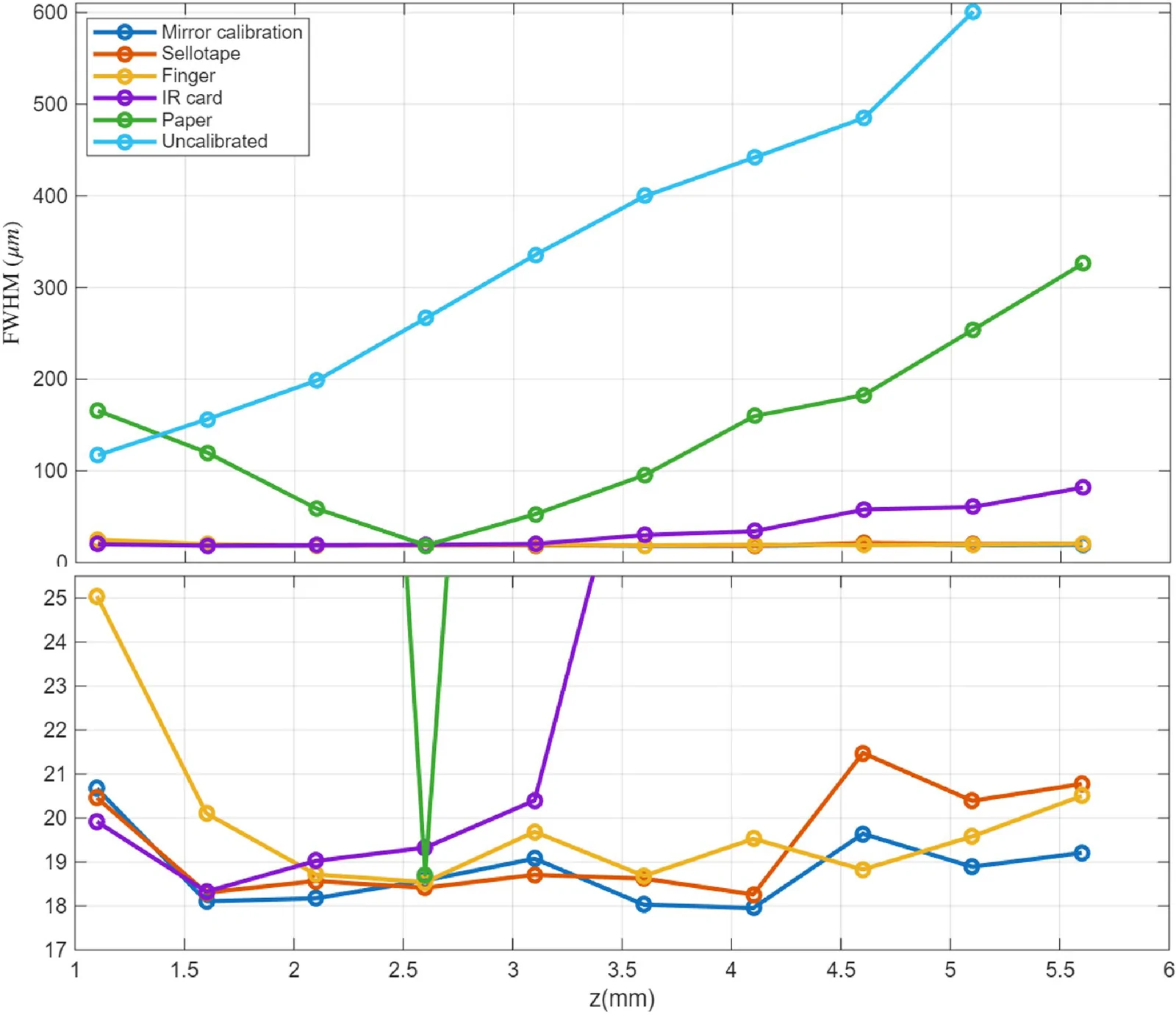
Axial resolution measured from reconstructed point spread functions as a function of imaging depth. The upper panel shows the full FWHM range. The lower panel shows an expanded view of the 17–26 μ m region, highlighting the differences between the mirror-derived calibration and the optimisation-derived calibrations. Representative reconstructed PSFs are provided in Supplement 1.

To evaluate the practical implications of these differences, PSFs were computed using both the reference and optimisation-derived functions (Fig. 6).

The sellotape phantom and *in vivo* finger datasets preserved axial resolution across most of the imaging range and remained closely matched to the mirror-calibrated PSFs. The IR card showed larger deviations at greater depths, while the paper dataset was accurate only near the dominant reflective interface.

Overall, the depth-dependent PSF measurements reflect the differences observed in the recovered calibration functions, with calibration performance becoming increasingly localised for samples containing more restricted axial structure.

## 5. Discussion

### 5.1. Image-driven calibration and identifiability

The presented results demonstrate that OCT calibration can be formulated as a data-driven optimisation problem operating directly on reconstructed image quality. Across all evaluated datasets, the optimisation framework consistently improved axial sharpness relative to the uncalibrated reconstructions and produced image quality comparable to conventional mirror-based calibration.

For samples containing reflectors distributed across a broad depth range, the recovered calibration functions closely matched the reference mirror-derived calibration. For the sellotape phantom, strong agreement was observed in both the calibration-function comparisons and depth-dependent PSF measurements, indicating that the recovered calibration generalised across the imaging range. The *in vivo* finger also produced calibration functions that generalised effectively across depth, although larger sample-dependent differences from the reference remained.

More substantial differences were observed for samples with more restricted axial structure. The IR card provides an intermediate example: its recovered calibration functions remained relatively close to the reference, but the corresponding PSFs progressively degraded at depths beyond the region occupied by the sample. This suggests that its more restricted axial distribution provided sufficient information to constrain the calibration within the observed depth range, but less strongly constrained its behaviour elsewhere. This behaviour was most pronounced for the paper dataset, which was dominated by a single reflective interface. The recovered calibration functions differed substantially from the reference, while accurate reconstruction was achieved only near the depth of the dominant interface.

Taken together, these results suggest that calibration recovery varies continuously with the information provided by the sample rather than representing a simple distinction between successful and unsuccessful optimisation. When reflectors span multiple depths, calibration parameters that improve one region while degrading another are penalised by the optimisation objective, providing stronger constraints towards a globally consistent calibration. Conversely, when structural information is confined to a narrow axial region, the optimisation problem becomes under-constrained, and multiple calibration solutions may produce similarly sharp local reconstructions.

This behaviour reflects an identifiability limit of recovering global calibration parameters from structurally limited image data. In this sense, the optimisation framework recovers the calibration information supported by the available sample structure, which may correspond either to a globally valid system calibration or to an image-specific effective calibration. This is consistent with the broader distinction between identifiability and estimability in inverse problems, where uniqueness and stability of the recovered solution depend on the information content of the observations [22–24]. This dependence on depth-distributed information is not unique to the image-driven formulation used here; conventional calibration similarly relies on measurements acquired across multiple OPDs to constrain the calibration functions globally.

### 5.2. Practical implications

Conventional OCT calibration methods can recover both wavenumber linearisation and dispersion compensation but typically rely on dedicated measurements of a reflector at multiple depths [4,5]. Other computational approaches address individual components of the calibration problem, such as dispersion compensation or numerical wavenumber correction [9,11]. Learning-based approaches have demonstrated correction of multiple sources of nonlinearity directly from OCT data. For example, Maliszewski *et al.* construct corrected A-scans by combining locally compensated regions selected by a trained neural network [12].

Our method differs by recovering explicit wavenumber remapping and dispersion compensation functions jointly from the image data itself, without requiring dedicated mirror measurements or prior network training. In the experiments presented here, a single B-scan of a simple multilayer phantom was sufficient to recover calibration functions that closely matched the conventional mirror-derived calibration and maintained axial resolution across the imaging range. This provides a simple route to software-defined calibration using a routine OCT image rather than a dedicated sequence of mirror measurements.

The dependence on sample structure determines how the recovered calibration should be interpreted in practice. For example, the IR card produced calibration functions that deviated from the reference at greater depths, while maintaining relatively good reconstruction performance over the depth range occupied by the sample. Such deviations could be of limited practical importance when imaging is restricted to the depth range represented within the optimisation data. In these cases, an image-specific effective calibration may be sufficient even if it does not reproduce the globally valid system calibration across the full axial range. However, applications requiring consistent resolution over a broad imaging depth require calibration data containing sufficient structural information across that depth range.

Beyond initial calibration, the framework may support several additional applications:

• **Calibration monitoring:** Previously measured calibration functions could be used to initialise the optimisation, allowing gradual system drift to be detected and corrected automatically.
• **Sample-specific dispersion compensation:** In systems with pre-linearised wavenumber sampling, the optimisation could be restricted to estimating only the dispersion function h(k) , enabling adaptive compensation directly from image data.
• **Clock-free operation:** With further development, the proposed approach could enable swept-source OCT operation without a dedicated k-clock, reducing system complexity and potentially lowering hardware costs by removing the need for clock circuitry and a clocked digitiser.
• **Volumetric calibration:** Extension to volumetric datasets may provide additional depth constraints and improve calibration robustness by incorporating richer structural information.

### 5.3. Limitations and future directions

Several limitations of the current framework warrant further investigation.

**Hyperparameter robustness.** The optimisation currently relies on fixed hyperparameters, including learning rates and loss weights. Although these parameters performed consistently across the datasets evaluated here, validation across a broader range of OCT system architectures remains necessary. Adaptive optimisation strategies may further improve robustness and reduce parameter tuning requirements.

**Loss function design.** The current loss uses image sparsity as a surrogate for calibration quality. Ideally, the image-quality objective would attain its optimum at the underlying system calibration, such that minimising the loss naturally guides the recovered functions towards the physically correct solution. In practice, this correspondence is not exact. For the datasets examined here, the optimisation can produce reconstructions with a lower sparsity-based loss than those obtained using the reference mirror calibration, despite the recovered functions differing from the reference. This indicates that the current objective can favour image-specific sharpening beyond that produced by the underlying system calibration.

The choice of image-quality objective therefore provides an additional constraint on the recovered solution alongside the structural information contained within the sample. Hoyer sparsity provided stable optimisation behaviour in the present experiments, but alternative objectives may produce different calibration solutions. Developing image-quality metrics that more strongly favour globally transferable calibration while remaining applicable to arbitrary OCT structure is therefore an important direction for future work.

**Sensitivity to image artifacts.** The optimisation assumes that dominant image features arise from true sample structure. Imaging artifacts such as fixed-pattern noise or motion-related distortions may therefore influence the recovered calibration. Incorporating preprocessing or artifact-aware regularisation could improve robustness in challenging acquisition conditions.

**Runtime and workflow integration.** The current implementation requires approximately 30–60 s to calibrate a single B-scan on a GPU. Although already practical for offline calibration, further acceleration could enable near real-time optimisation directly during image acquisition. In such a workflow, the optimisation would function as an image-specific correction procedure, adapting reconstruction quality to the current sample and imaging conditions rather than recovering a globally optimal system calibration. This may provide a flexible alternative to conventional calibration procedures in applications where rapid setup and adaptive correction are prioritised.

## 6. Conclusion

We have presented a physics-informed optimisation framework for OCT calibration directly from a single B-scan. By jointly estimating wavenumber remapping and dispersion compensation within the CLF reconstruction framework, the method recovers calibration functions capable of producing image quality comparable to conventional mirror-based calibration without requiring dedicated calibration acquisitions.

The results demonstrate that successful recovery of globally consistent calibration depends strongly on the structural diversity present within the optimisation dataset. Samples containing reflectors distributed across depth enabled recovery of calibration functions closely matching the reference mirror-derived calibration, while structurally limited samples produced image-specific effective calibration primarily within the observed depth range.

These findings show that OCT calibration can be formulated as an inverse imaging problem constrained by sample structure rather than solely as a hardware calibration procedure. With further optimisation and validation, such approaches may support more flexible, software-driven, and adaptive OCT calibration workflows.

## Supplemental information

Supplement 1 This file contains all of the supplementary figures alongside some descriptive text https://doi.org/10.6084/m9.figshare.33273870

## Data Availability

Data underlying the results presented in this paper are not publicly available at this time but may be obtained from the authors upon reasonable request.

## Supplemental document

See Supplement 1 for supporting content.
