# Supplementary material for: Image-driven OCT calibration from a single B-scan
Source: Biomed Opt Express. 2026 Aug 25;17(9):4778–94. doi: 10.1364/BOE.608117 (PMC13592270; doi:10.1364/BOE.608117)
Supplement: Supplementary file 1 [file boe-17-9-4778-s001.pdf]

Supplemental Document

Biomedical Optics EXPRESS

Image-driven OCT calibration from a single B-scan: supplement

OWEN SIMMONS,[1,*] PHILIPPE DE WILDE,[2] AND ADRIAN PODOLEANU[1]

[1]*Applied Optics Group, University of Kent, Canterbury, Kent, CT2 7NH, UK*
[2]*Division of Natural Sciences, University of Kent, Canterbury, Kent, CT2 7NH, UK*
[*]*os343@kent.ac.uk*

This supplement published with Optica Publishing Group on 25 August 2026 by The Authors under the terms of the Creative Commons Attribution 4.0 License in the format provided by the authors and unedited. Further distribution of this work must maintain attribution to the author(s) and the published article's title, journal citation, and DOI.

Supplement DOI: https://doi.org/10.6084/m9.figshare.33273870

Parent Article DOI: https://doi.org/10.1364/BOE.608117

1 Loss curves

Figure S1 shows the total loss during optimisation for each dataset. The staged optimisation strategy produces a consistent decrease in loss, with plateaus occurring prior to each spline refinement stage. Small increases can be observed immediately after refinement as newly introduced control points are optimised.

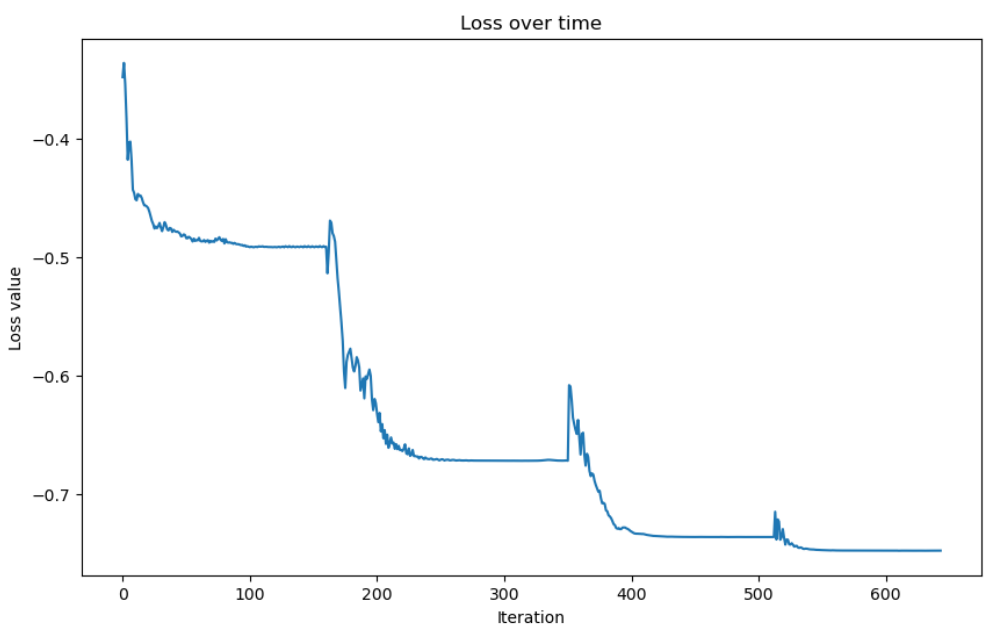

(a) Sellotape phantom

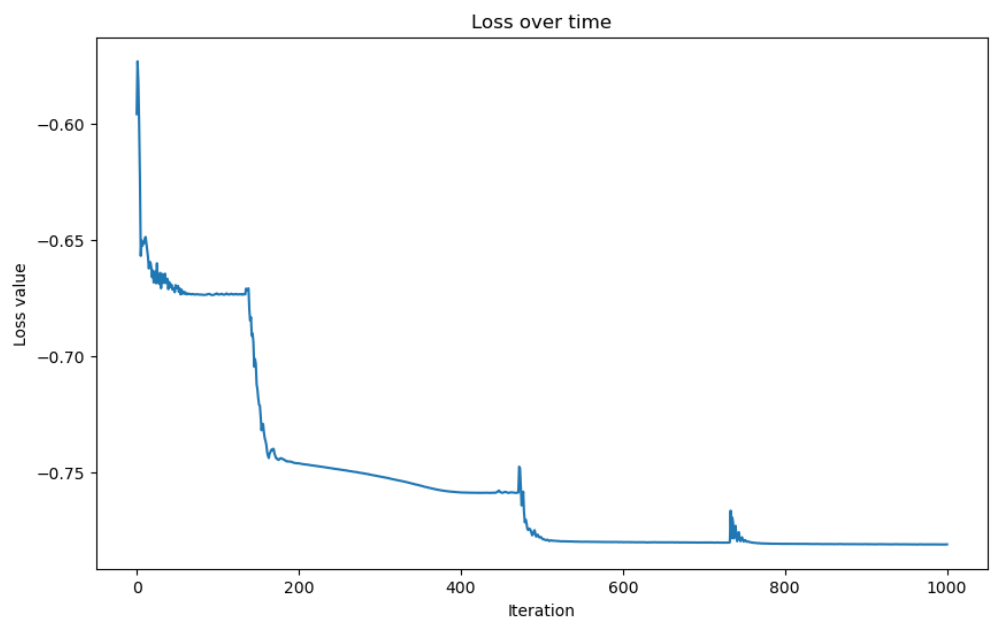

(b) Infrared card

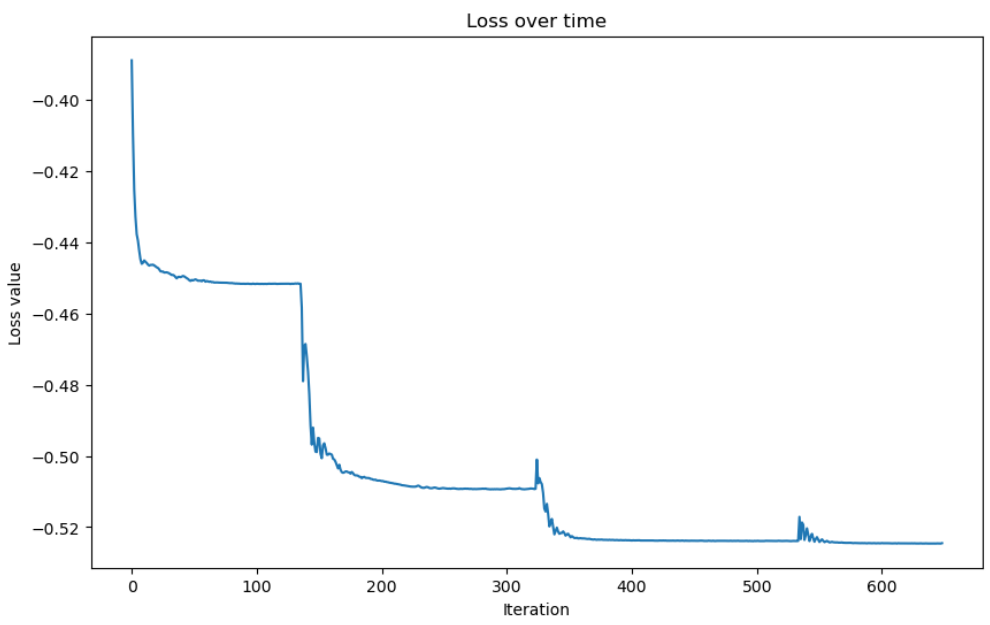

(c) *In vivo* finger

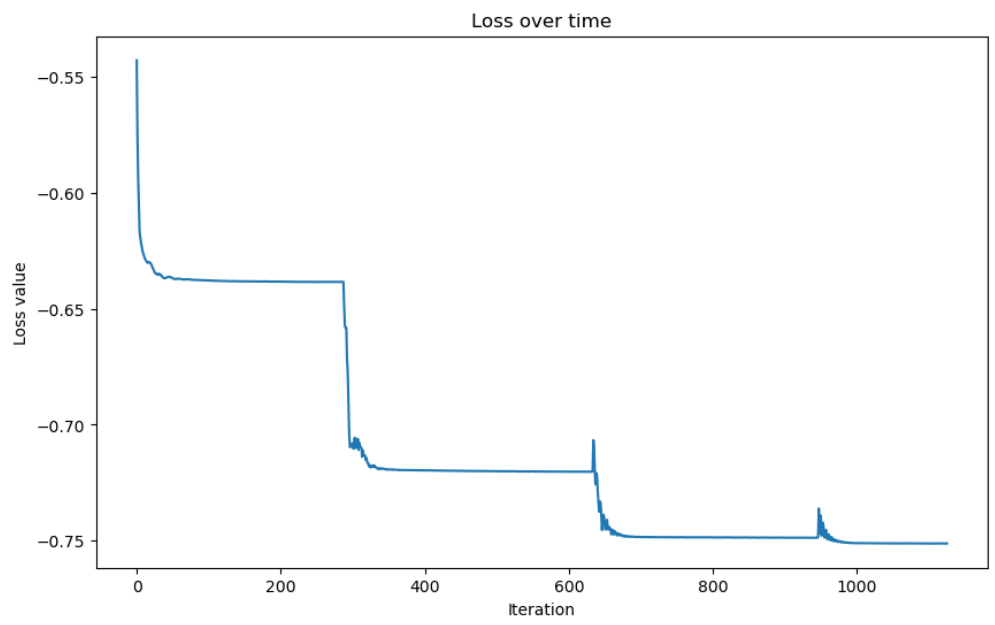

(d) Paper

Figure S1: Optimisation loss as a function of iteration for each dataset.

2 Calibration function comparisons

Figures S2–S5 show the recovered calibration functions for all datasets. The sellotape, IR card and finger datasets produce calibration functions that closely match the mirror-derived reference. The paper dataset exhibits substantial deviations, particularly in the recovered dispersion function. The $g(k)$ and $h(k)$ functions are less sensitive to differences than the gradients shown in the main manuscript.

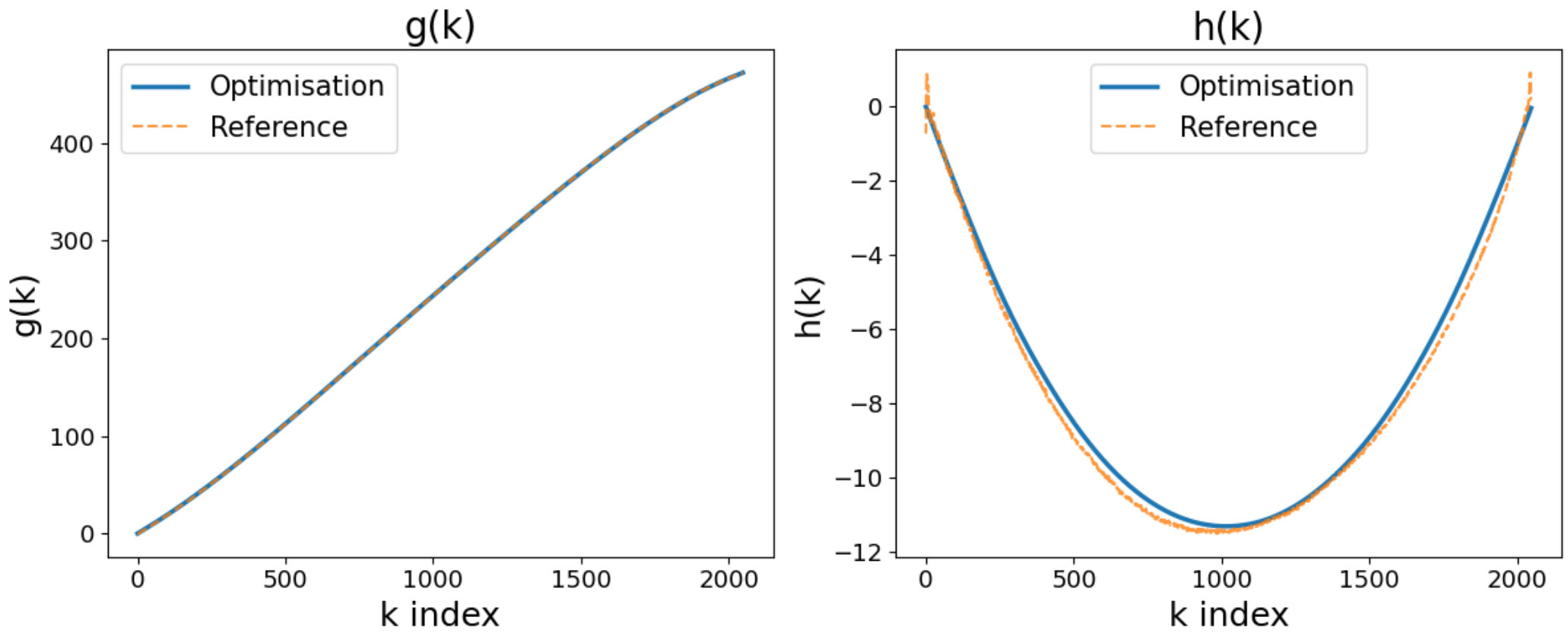

Figure S2: Recovered calibration functions $g(k)$, $h(k)$ for the sellotape phantom dataset.

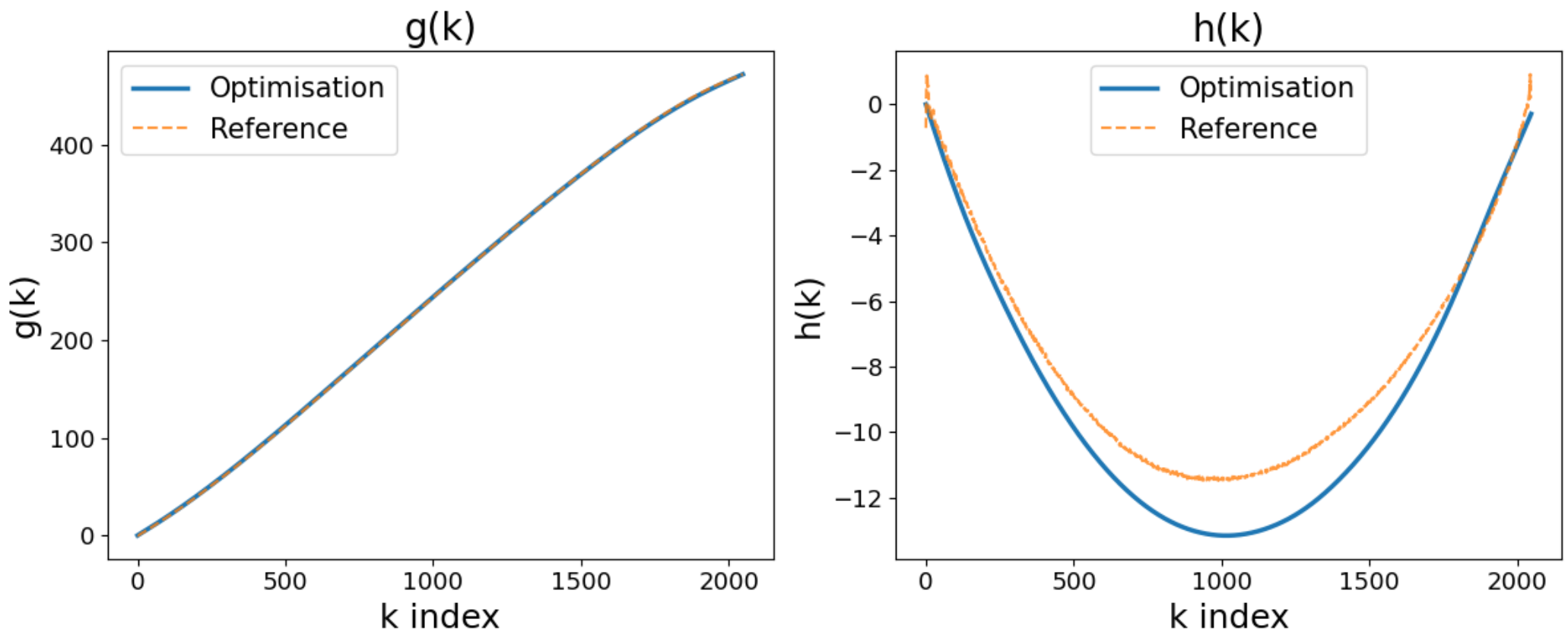

Figure S3: Recovered calibration functions $g(k)$, $h(k)$ for the IR card dataset

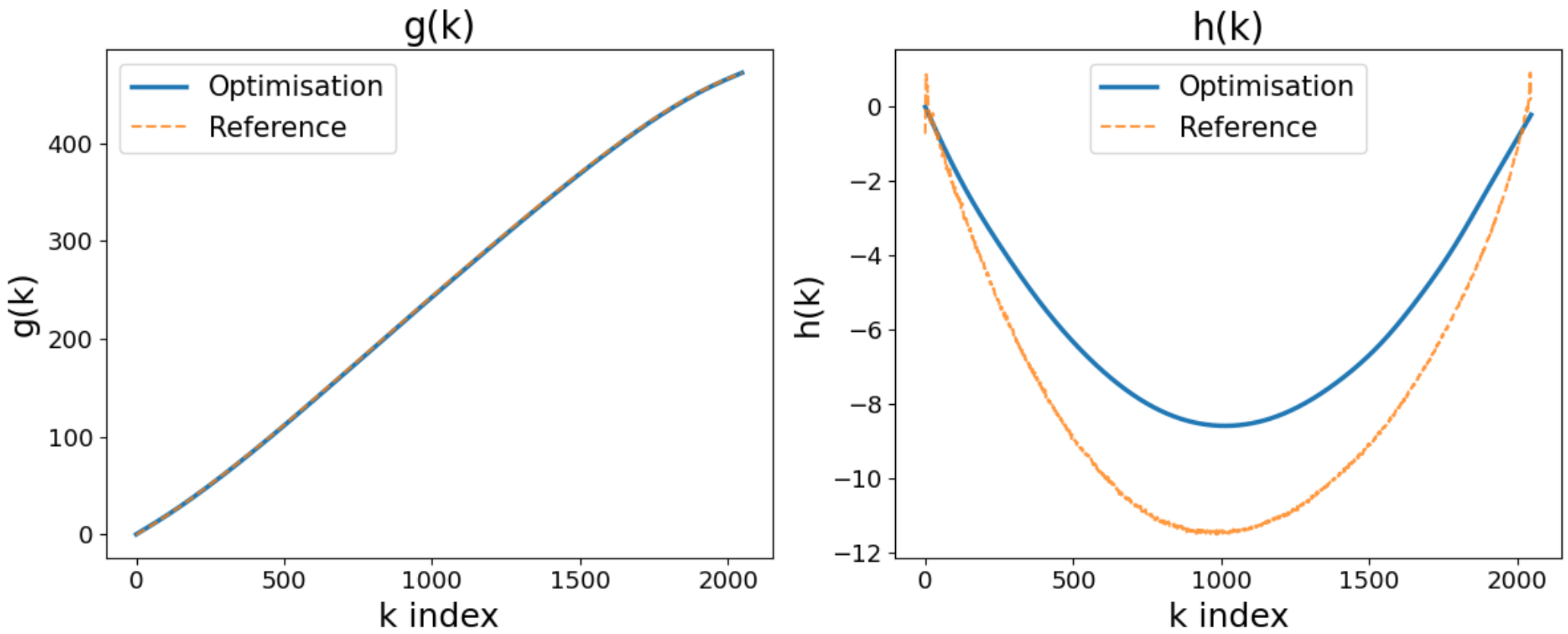

Figure S4: Recovered calibration functions $g(k)$, $h(k)$ for the *in vivo* finger dataset

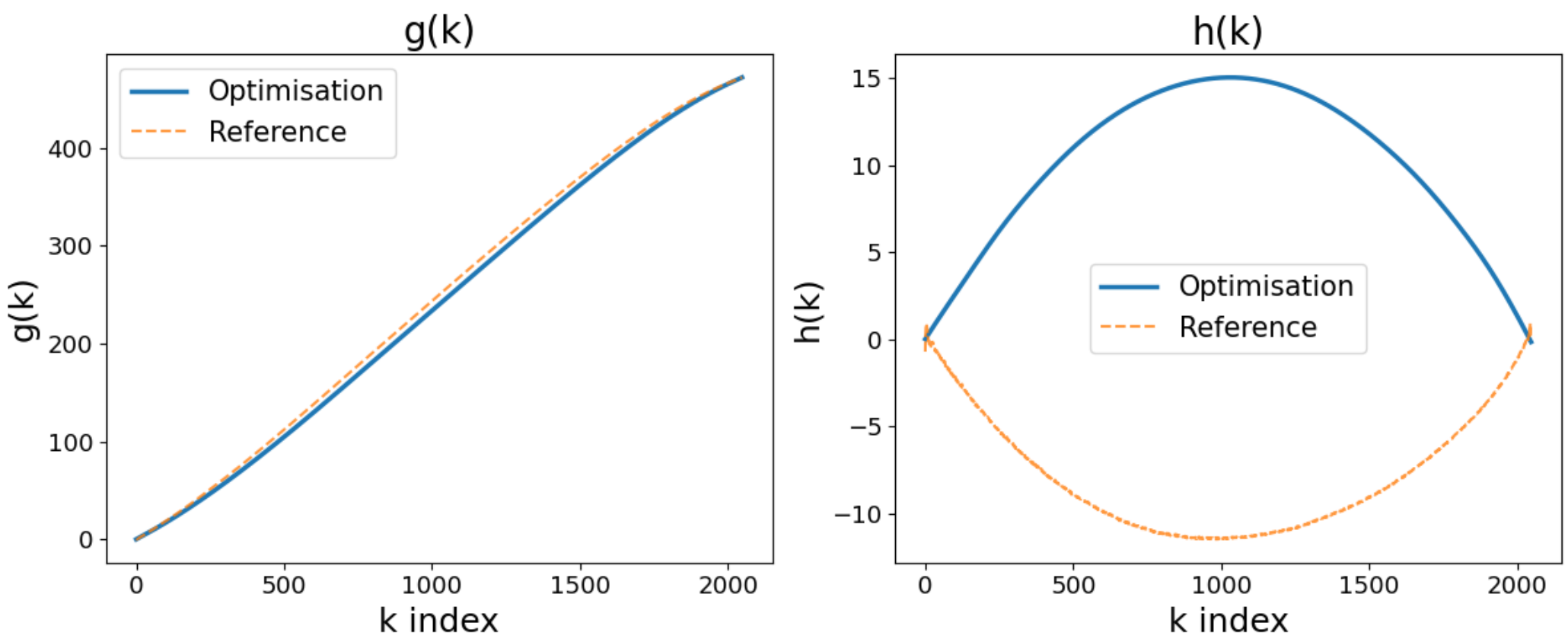

Figure S5: Recovered calibration functions $g(k)$, $h(k)$ for the paper dataset

Table 1: Normalised Root Mean Square Error and Cosine similarity between the optimisation-derived and reference calibration functions.

**Sample**	**NRMSE** $g(k)$	**NRMSE** $h(k)$	**CosSim** $g(k)$	**CosSim** $h(k)$
Sellotape	0.00165	0.0925	1.000	0.998
IR card	0.00486	0.347	1.000	0.998
Finger	0.00632	0.680	1.000	0.997
Paper	0.0502	5.775	1.000	-0.998

3 Transferability of calibration

Figures S6–S9 demonstrate the transferability of calibration functions recovered from each dataset. Calibrations derived from the sellotape, infrared card and finger datasets generalise well to other samples, producing image quality comparable to the reference calibration. Some mis-calibration is evident at the extremes of the tape image for the IR card. In contrast, the calibration recovered from the paper dataset performs poorly when applied to other samples. This behaviour is consistent with the identifiability discussion presented in Section 5 of the main text.

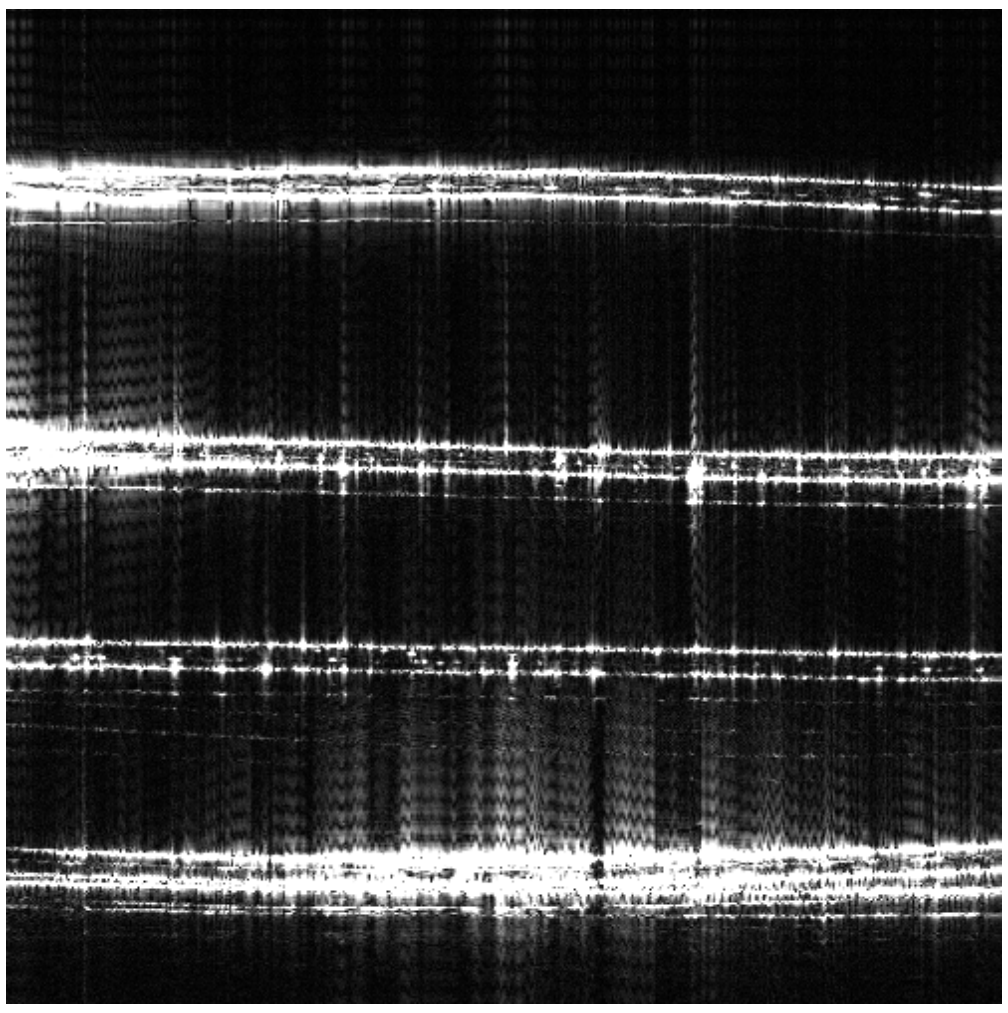

(a) Sellotape

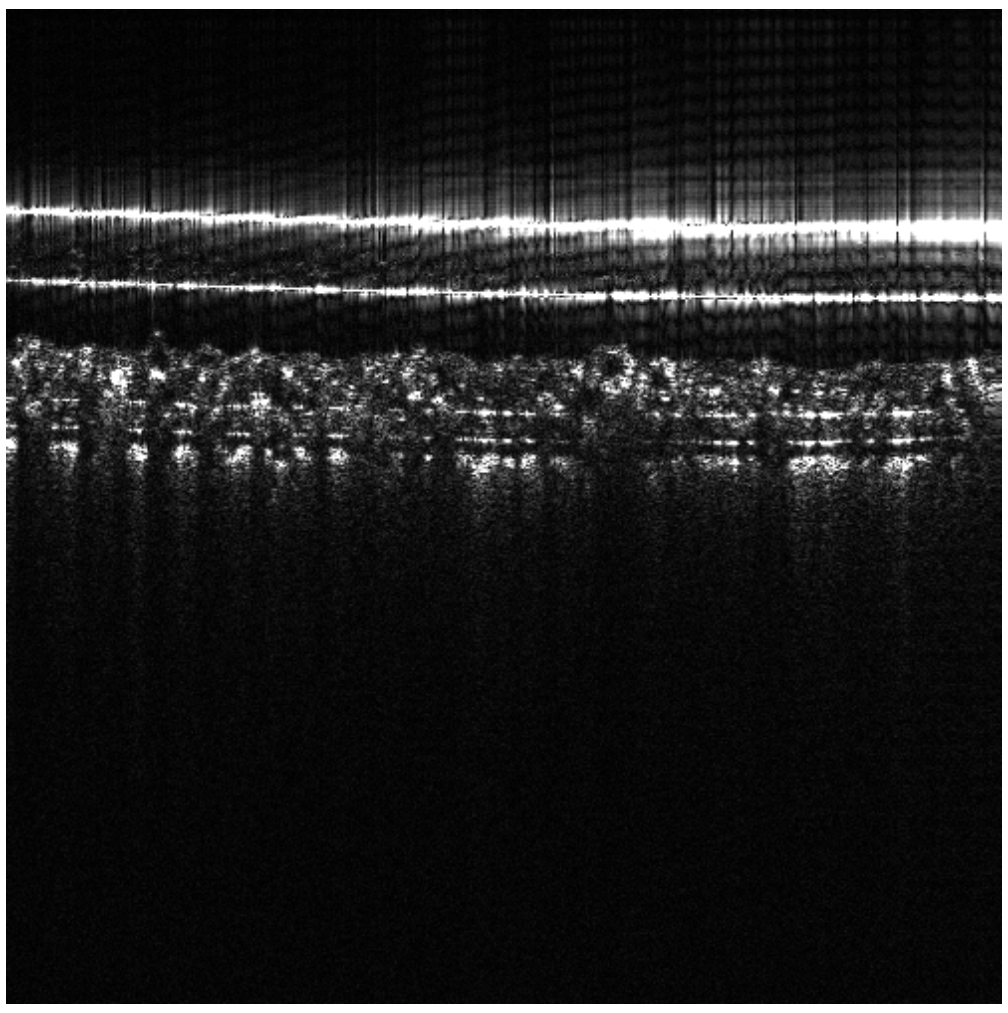

(b) IR card

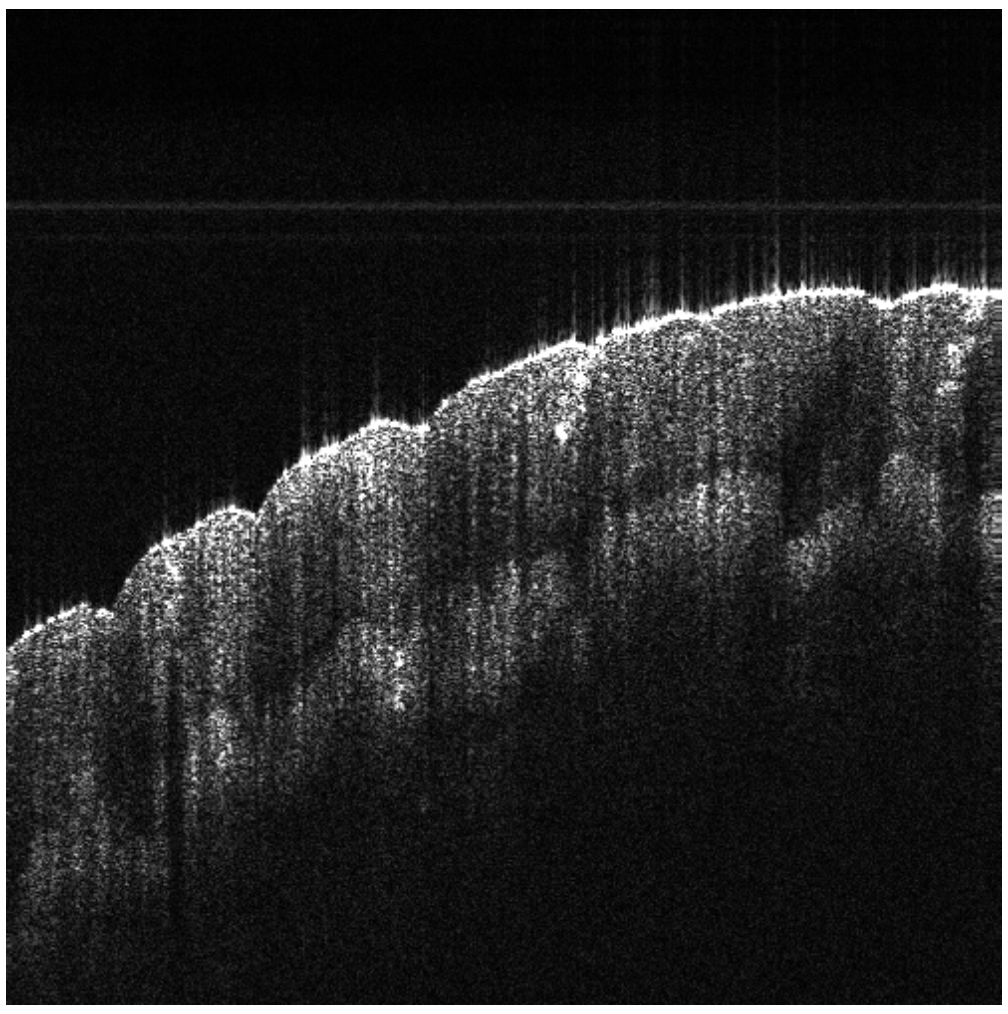

(c) Finger

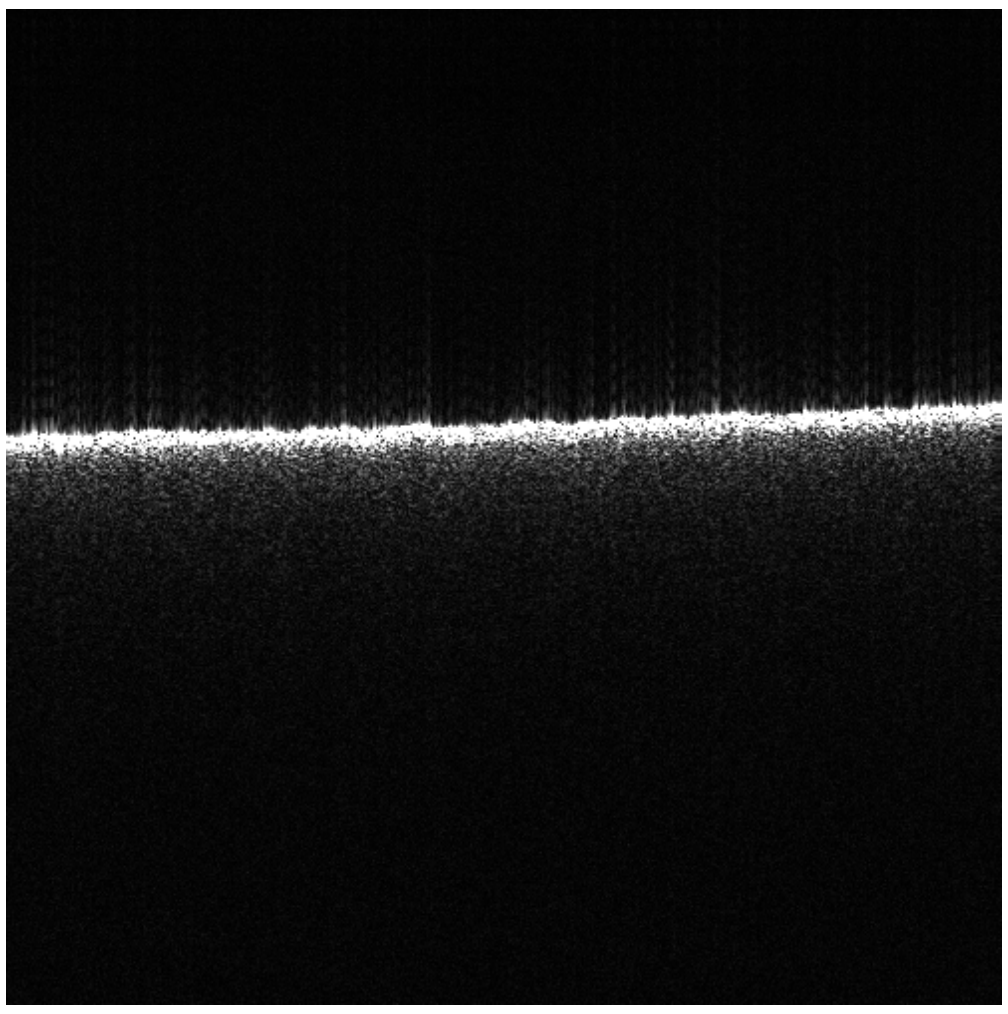

(d) Paper

Figure S6: Each sample produced with calibration from sellotape

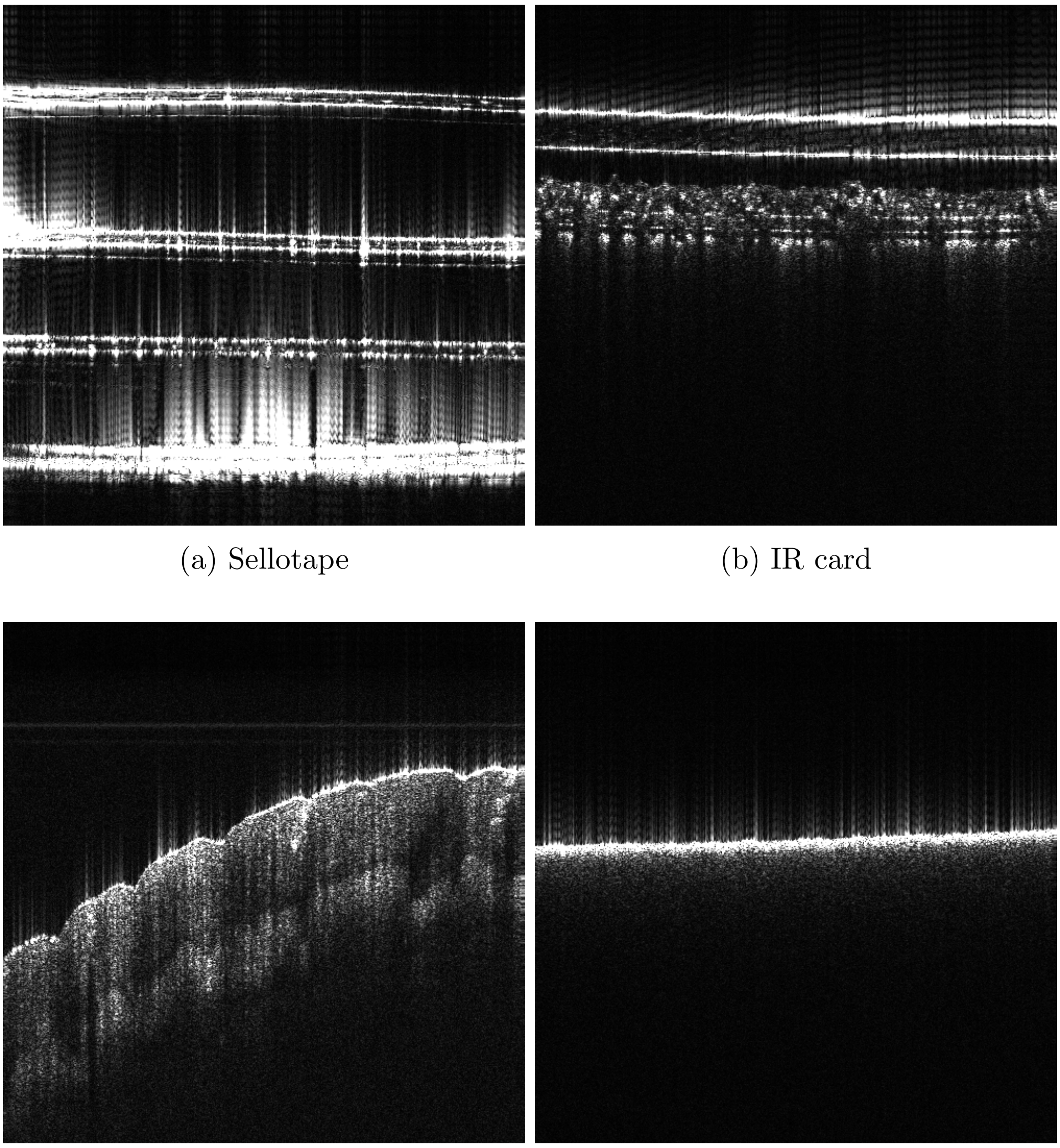

(a) Sellotape

(b) IR card

(c) Finger

(d) Paper

Figure S7: Each sample produced with calibration from IR card

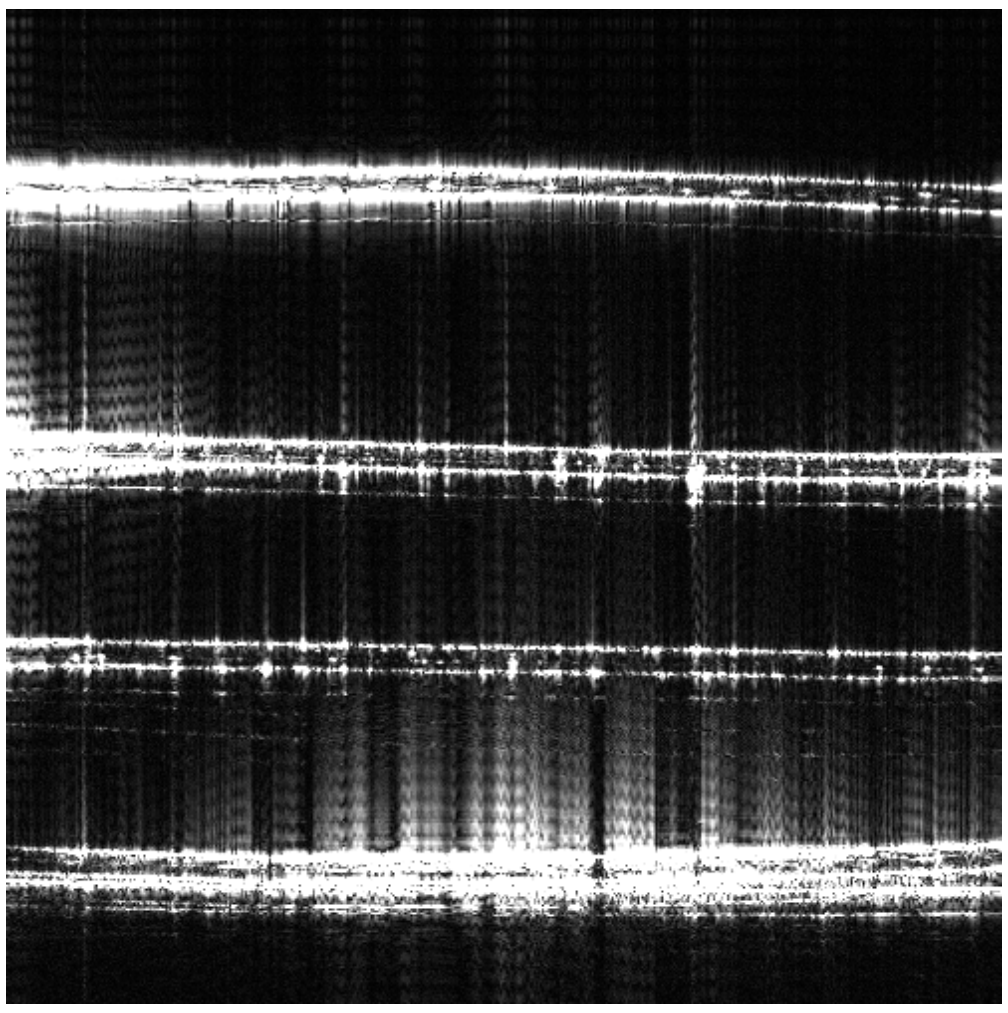

(a) Sellotape

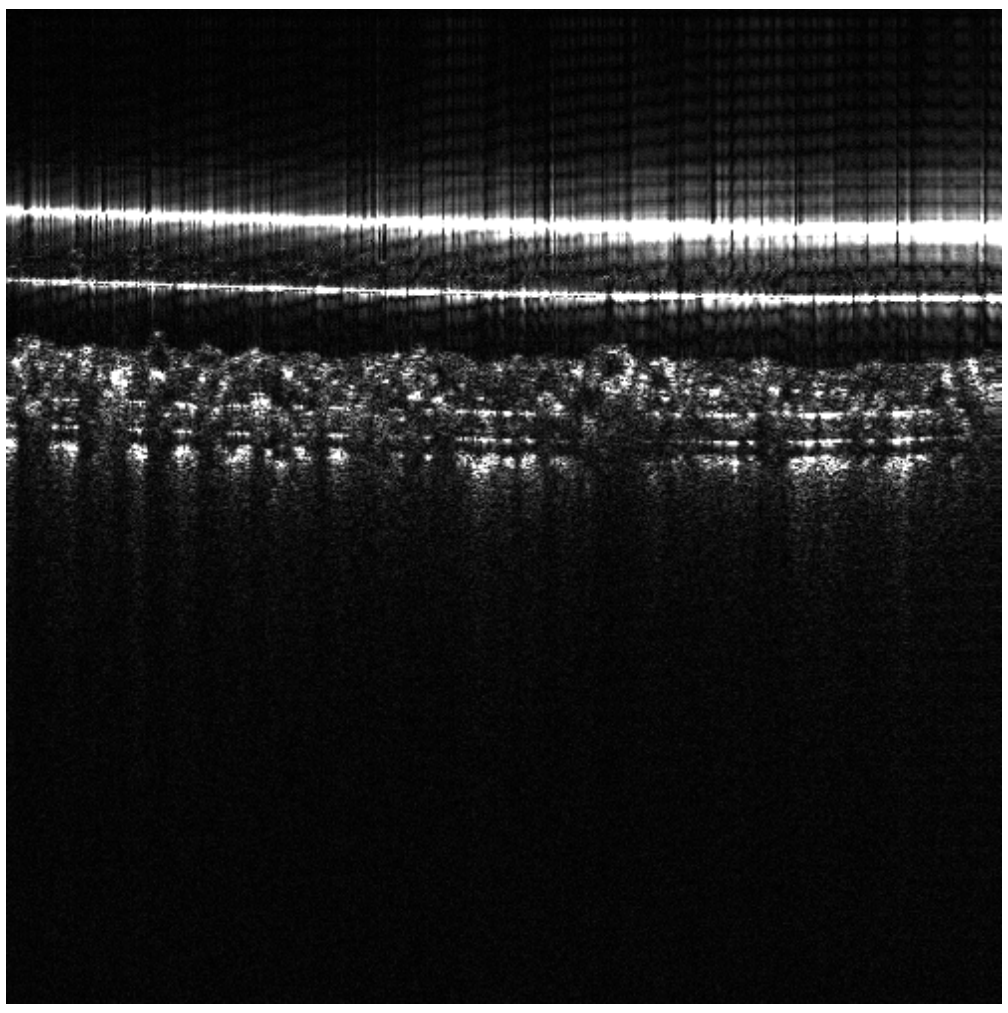

(b) IR card

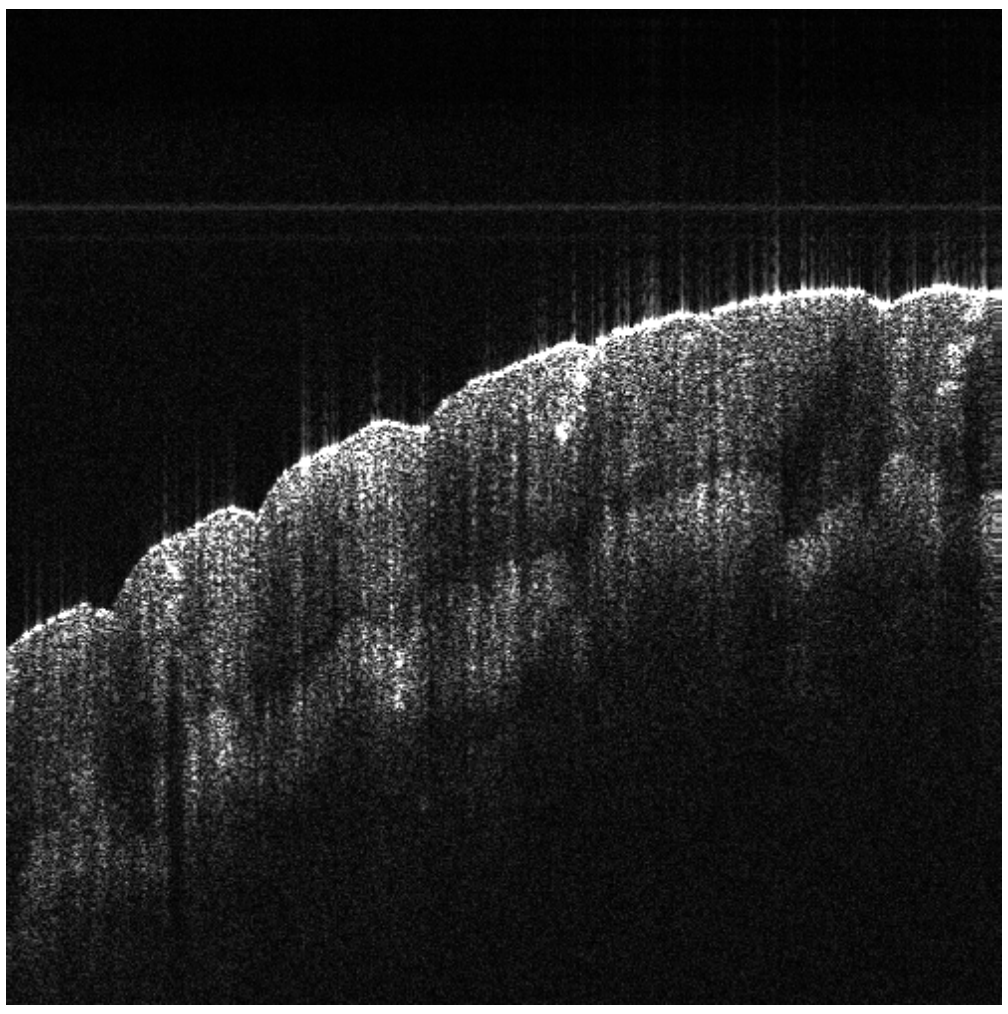

(c) Finger

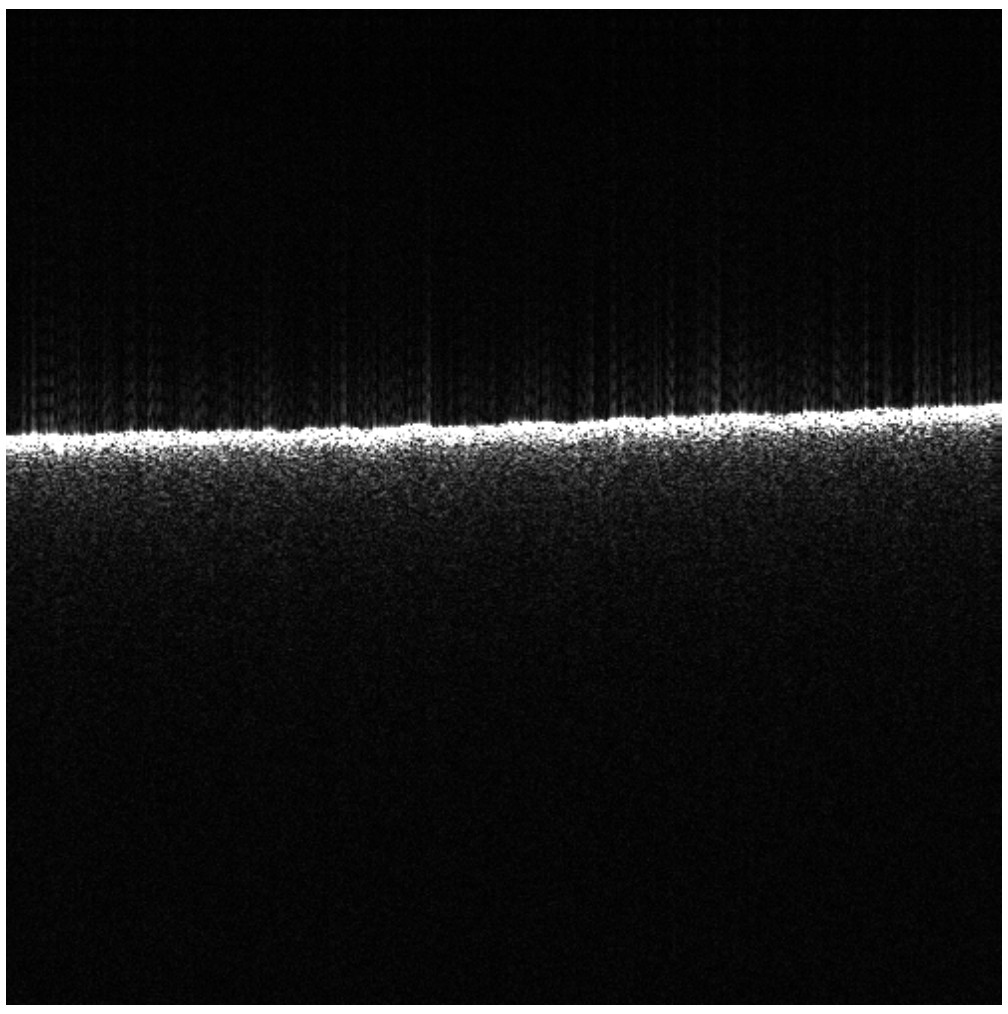

(d) Paper

Figure S8: Each sample produced with calibration from *in vivo* finger

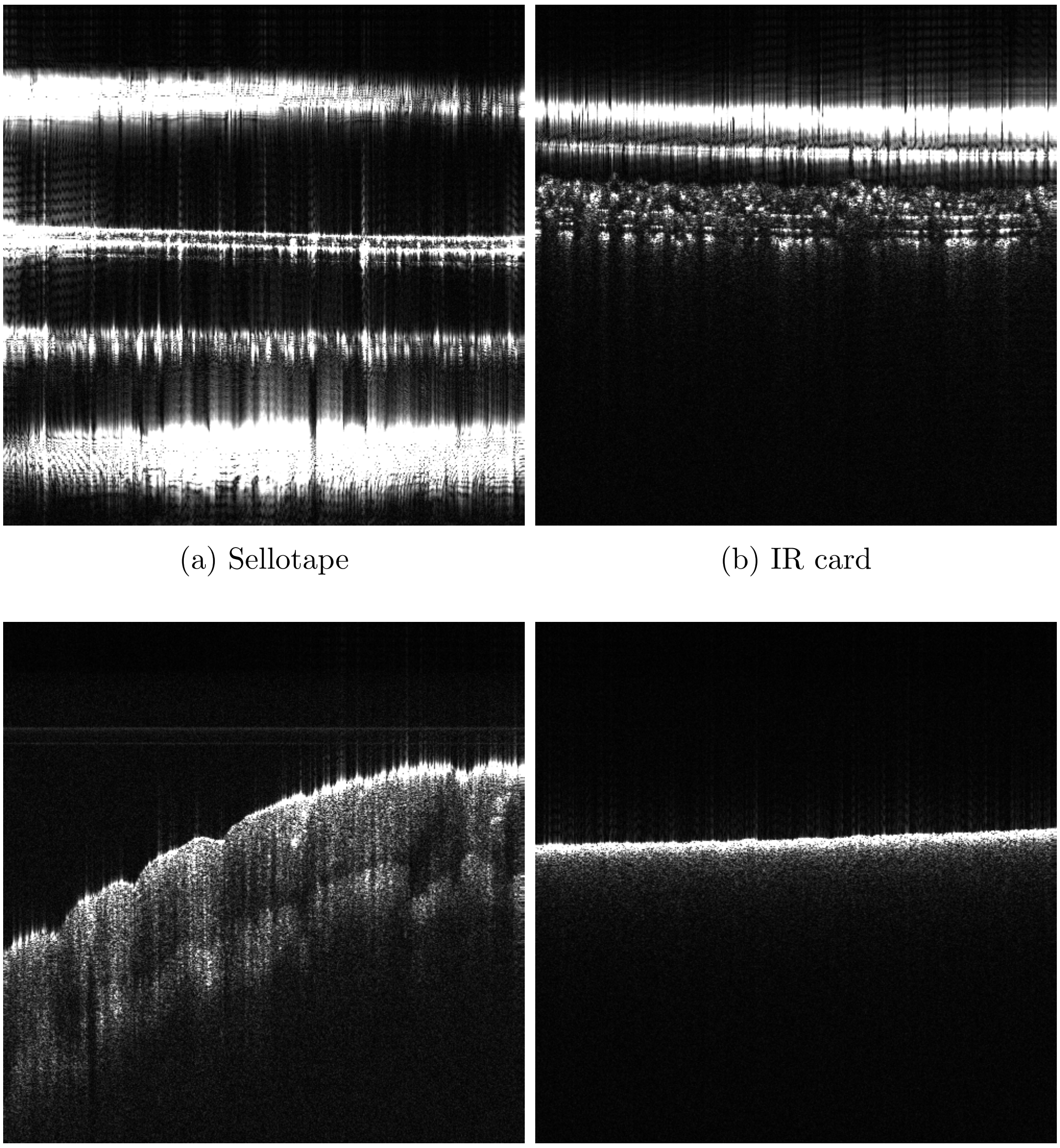

(a) Sellotape

(b) IR card

(c) Finger

(d) Paper

Figure S9: Each sample produced with calibration from paper

4 PSFs

Figure S10 shows the reconstructed point spread functions (PSFs) used to generate the depth-dependent FWHM measurements reported in Fig. 6 of the main text. Each plot contains PSFs reconstructed at multiple optical path differences using the corresponding calibration function.

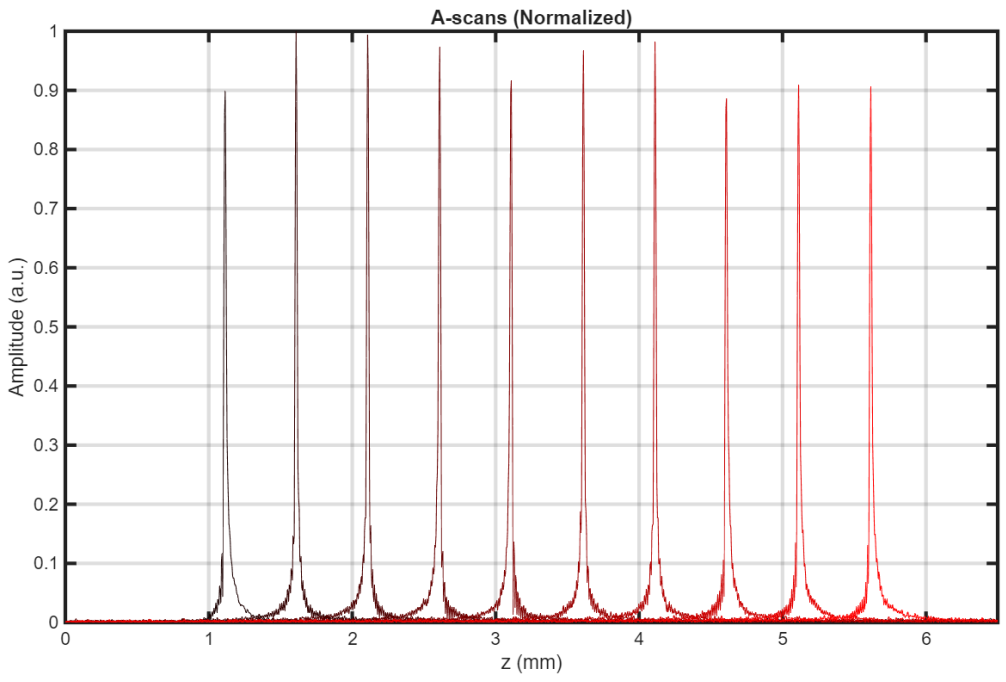

(a) Mirror calibration

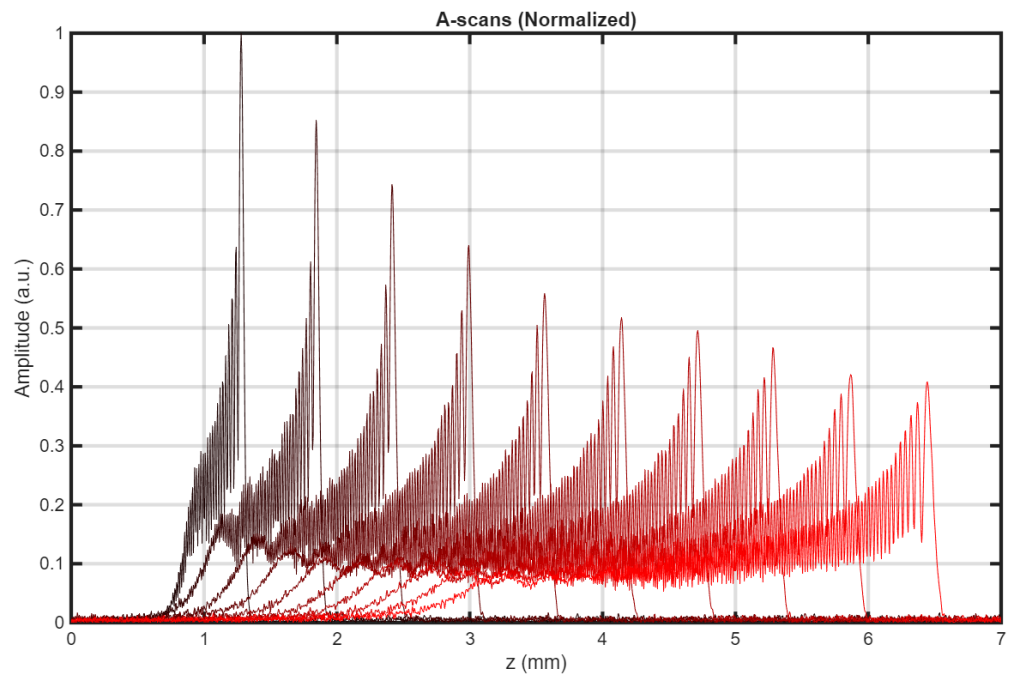

(b) Uncalibrated

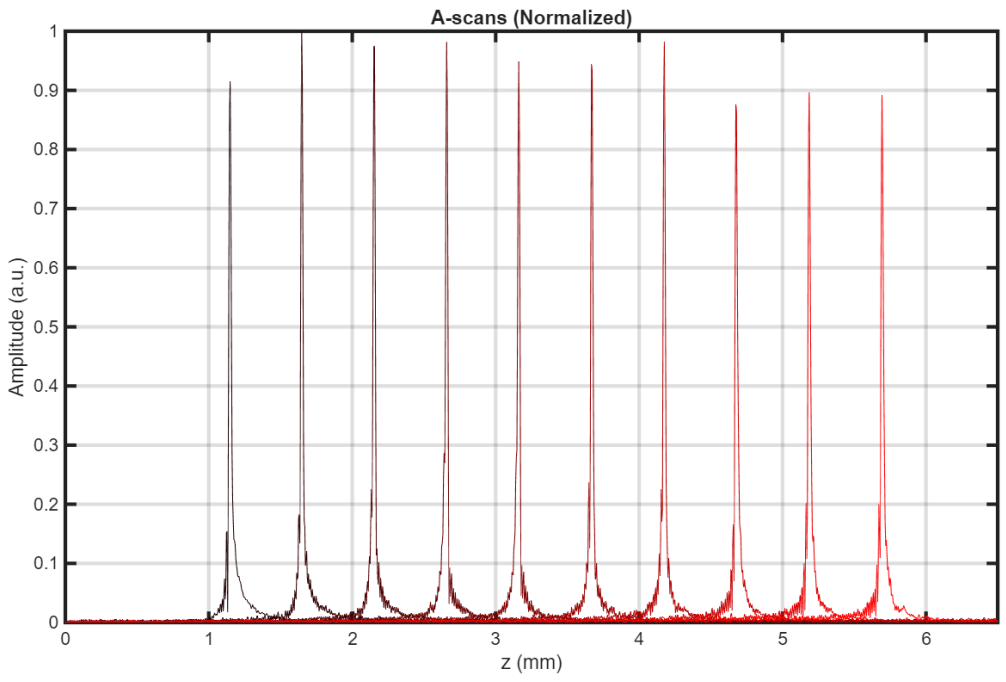

(c) Sellotape

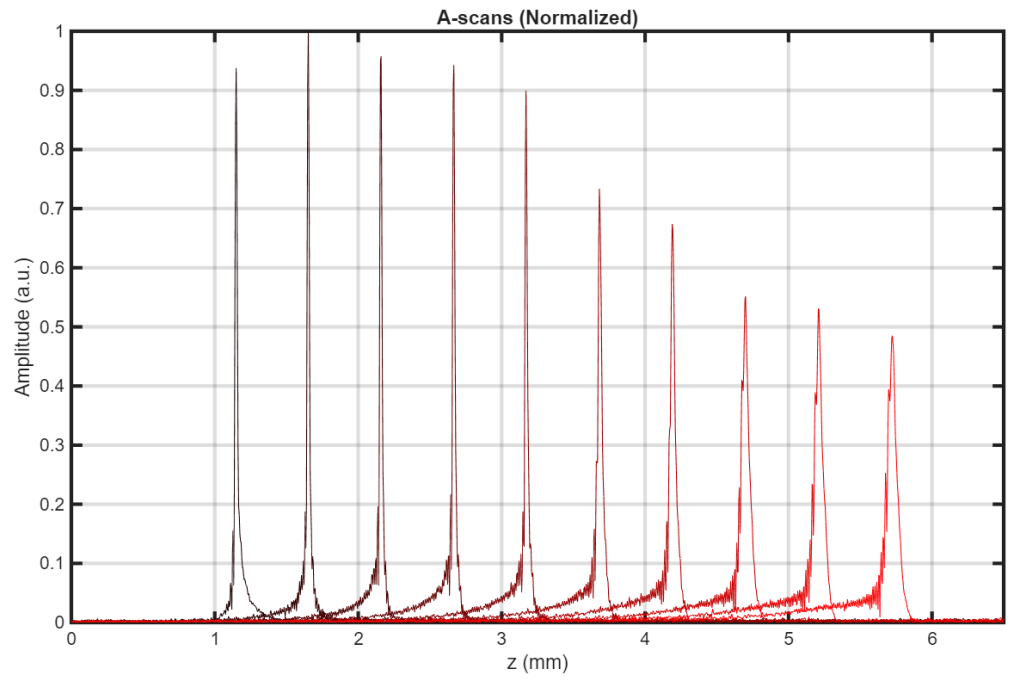

(d) IR card

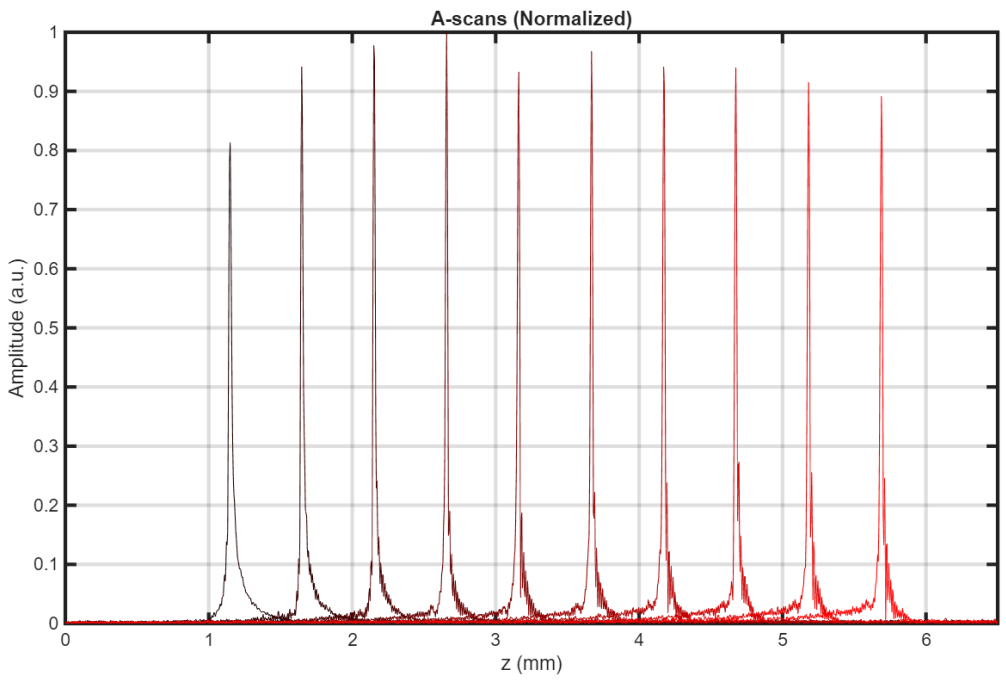

(e) *In vivo* finger

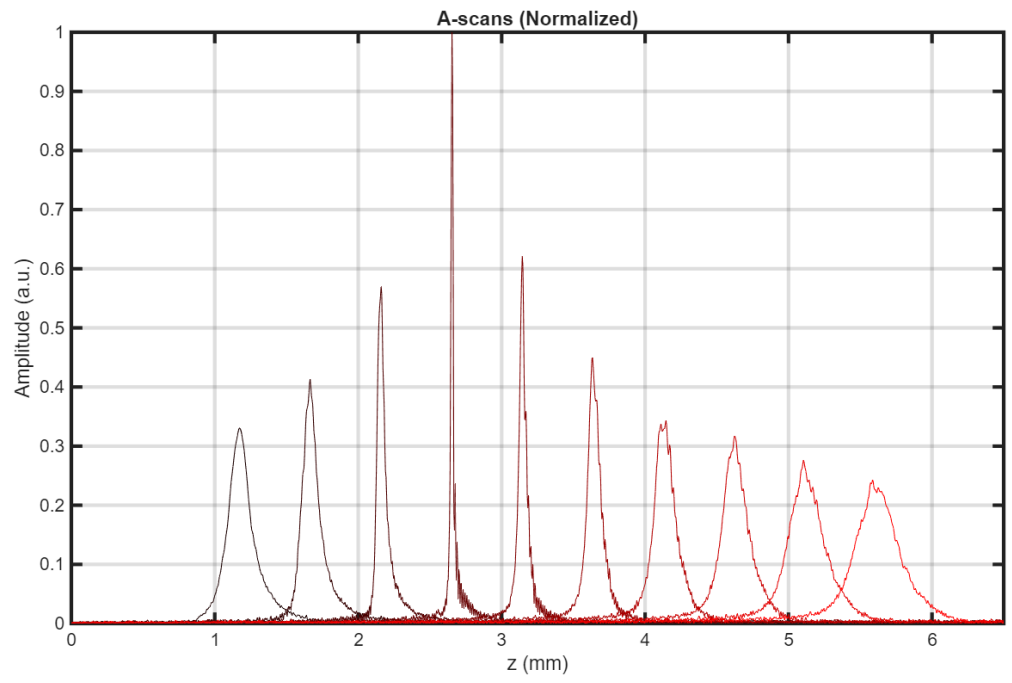

(f) Paper

Figure S10: Reconstructed point spread functions used to calculate the depth-dependent axial resolution measurements shown in Fig. 6 of the main manuscript.
